# Supplementary material for: Light contamination in stable isotope-labelled internal peptide standards is frequent and a potential source of false discovery and quantitation error in proteomics
Source: Anal Bioanal Chem. 2022 Feb 4;414(8):2545–52. doi: 10.1007/s00216-022-03931-w (PMC8888373; doi:10.1007/s00216-022-03931-w)

Table S1. Full list of peptides acquired in this study and the assessed ppm quantitation for the light contamination (see separate Excel file)

Peptide numbering reflects the position in Figure 1c. Vendor information is given as specified in the experimental section. Light contamination signal is given as fraction of light precursor peak area vs heavy precursor peak area. Separate columns list measurements where peptide contaminations were confirmed individually as opposed to bulk measurement in mixture.

Figure S1. Full set of extracted ion chromatograms acquired for light contamination assessment.

Extracted ion chromatograms for heavy (bottom) and light (top) precursors. Where appropriate, panels showing peptide chromatograms from single peptide solutions are given next to chromatograms acquired from the full peptide mixture. dotp and ppm assessment of heavy vs light is calculated from the summed peak area of the top 4 transitions of the displayed chromatograms. Where light contamination signal was not selected as identified, ppm and dotp are given as “NA” (not available).

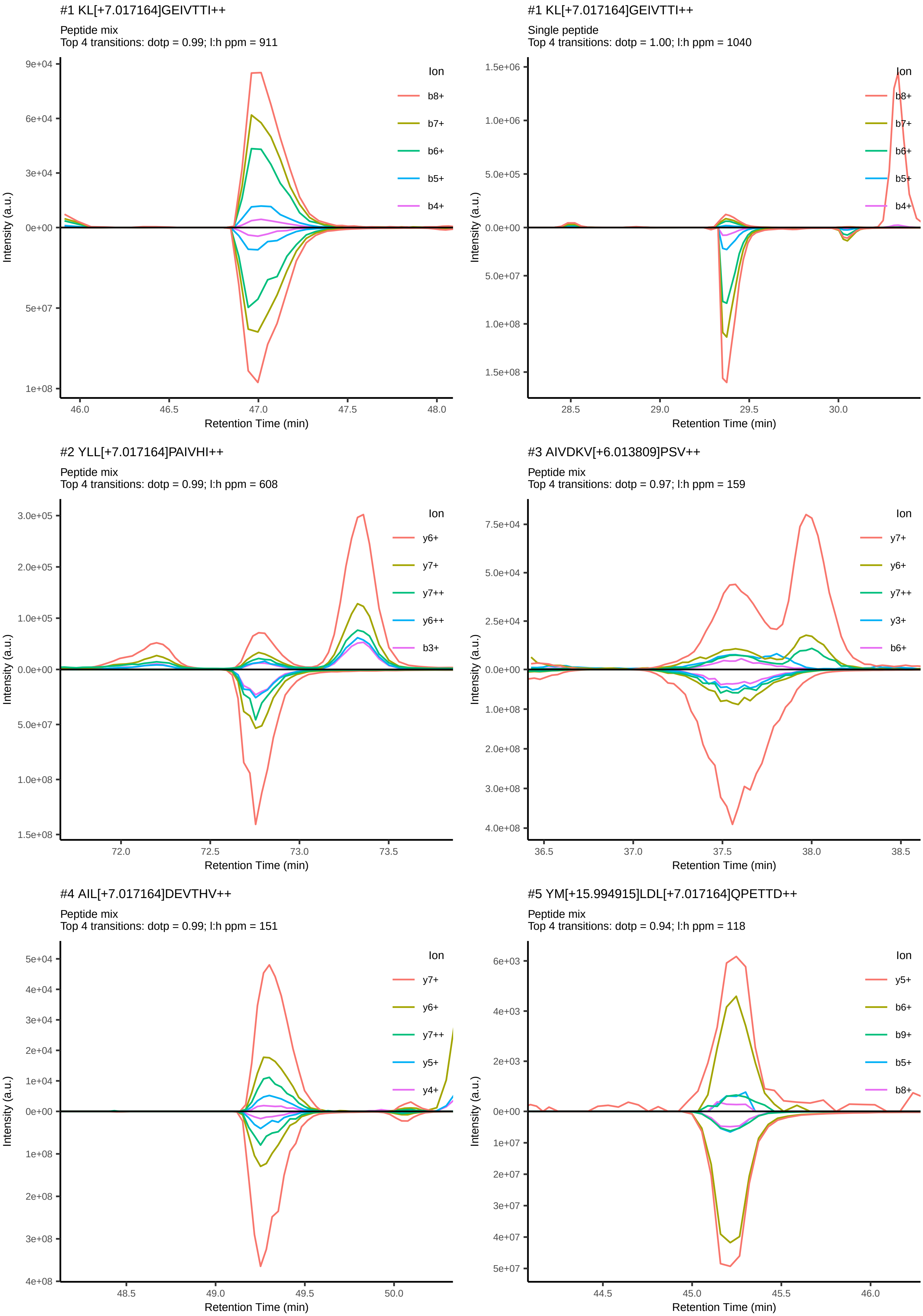
#1 KL[+7.017164]GEIVTTI++
Peptide mix
Top 4 transitions: dotp = 0.99; l:h ppm = 911
Ion
b8+
b7+
b6+
b5+
b4+
9e+04
6e+04
3e+04
0e+00
5e+07
1e+08
46.0
46.5
47.0
47.5
48.0
Intensity (a.u.)
Retention Time (min)
#1 KL[+7.017164]GEIVTTI++
Single peptide
Top 4 transitions: dotp = 1.00; l:h ppm = 1040
1.5e+06
1.0e+06
5.0e+05
0.0e+00
5.0e+07
1.0e+08
1.5e+08
28.5
29.0
29.5
30.0
#2 YLL[+7.017164]PAIVHI++
Peptide mix
Top 4 transitions: dotp = 0.99; l:h ppm = 608
Ion
y6+
y7+
y7++
y6++
b3+
3.0e+05
2.0e+05
1.0e+05
0.0e+00
5.0e+07
1.0e+08
1.5e+08
72.0
72.5
73.0
73.5
#3 AIVDKV[+6.013809]PSV++
Peptide mix
Top 4 transitions: dotp = 0.97; l:h ppm = 159
Ion
y7+
y6+
y7++
y3+
b6+
7.5e+04
5.0e+04
2.5e+04
0.0e+00
1.0e+08
2.0e+08
3.0e+08
4.0e+08
36.5
37.0
37.5
38.0
38.5
#4 AIL[+7.017164]DEVTHV++
Peptide mix
Top 4 transitions: dotp = 0.99; l:h ppm = 151
Ion
y7+
y6+
y7++
y5+
y4+
5e+04
4e+04
3e+04
2e+04
1e+04
0e+00
1e+08
2e+08
3e+08
4e+08
48.5
49.0
49.5
50.0
#5 YM[+15.994915]LDL[+7.017164]QPETTD++
Peptide mix
Top 4 transitions: dotp = 0.94; l:h ppm = 118
Ion
y5+
b6+
b9+
b5+
b8+
6e+03
4e+03
2e+03
0e+00
1e+07
2e+07
3e+07
4e+07
5e+07
44.5
45.0
45.5
46.0

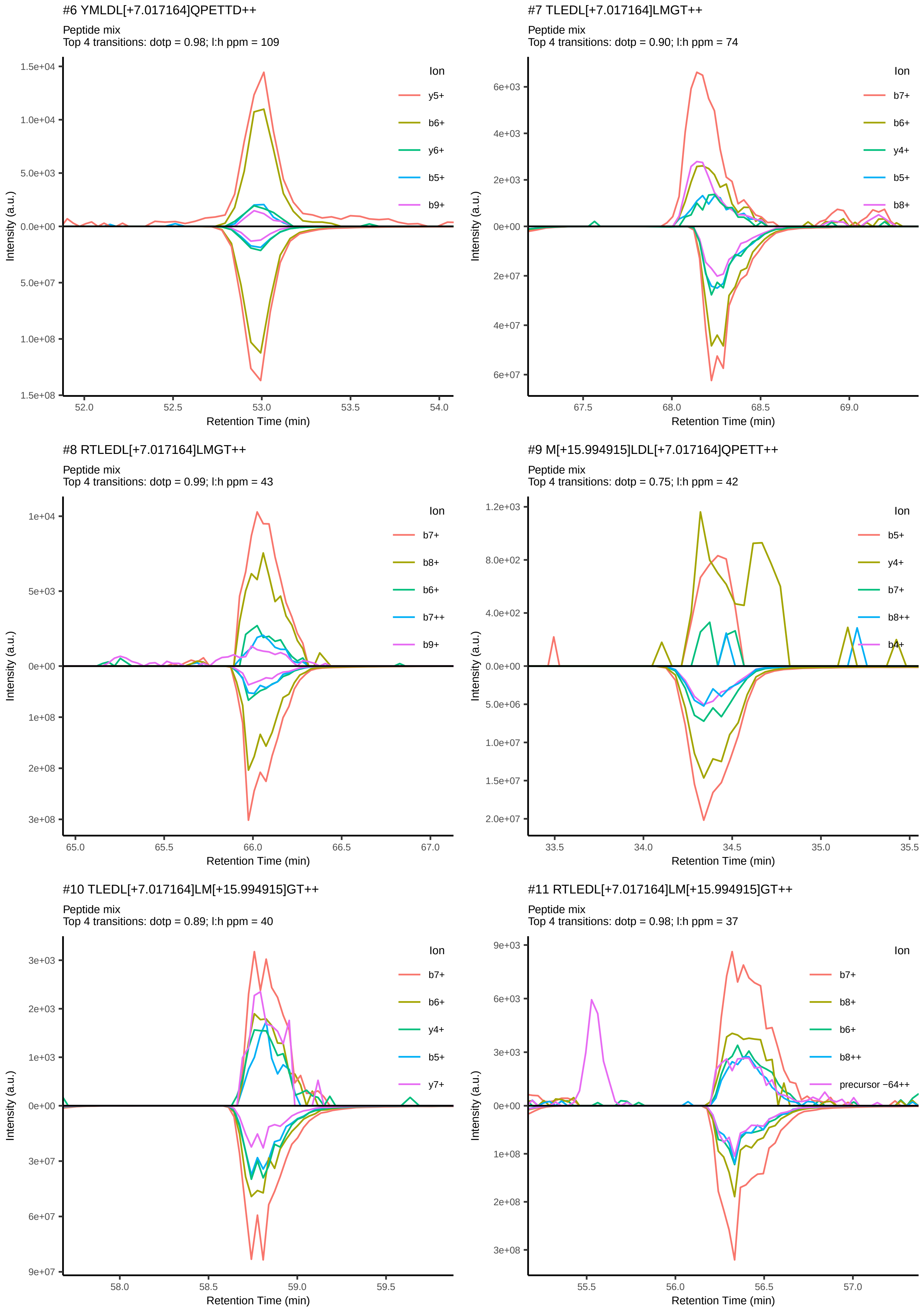
#6 YMLDL[+7.017164]QPETTD++
Peptide mix
Top 4 transitions: dotp = 0.98; l:h ppm = 109
Ion
y5+
b6+
y6+
b5+
b9+
Intensity (a.u.)
1.5e+04
1.0e+04
5.0e+03
0.0e+00
5.0e+07
1.0e+08
1.5e+08
52.0
52.5
53.0
53.5
54.0
Retention Time (min)
#7 TLEDL[+7.017164]LMGT++
Peptide mix
Top 4 transitions: dotp = 0.90; l:h ppm = 74
Ion
b7+
b6+
y4+
b5+
b8+
Intensity (a.u.)
6e+03
4e+03
2e+03
0e+00
2e+07
4e+07
6e+07
67.5
68.0
68.5
69.0
Retention Time (min)
#8 RTLEDL[+7.017164]LMGT++
Peptide mix
Top 4 transitions: dotp = 0.99; l:h ppm = 43
Ion
b7+
b8+
b6+
b7++
b9+
Intensity (a.u.)
1e+04
5e+03
0e+00
1e+08
2e+08
3e+08
65.0
65.5
66.0
66.5
67.0
Retention Time (min)
#9 M[+15.994915]LDL[+7.017164]QPETT++
Peptide mix
Top 4 transitions: dotp = 0.75; l:h ppm = 42
Ion
b5+
y4+
b7+
b8++
b4+
Intensity (a.u.)
1.2e+03
8.0e+02
4.0e+02
0.0e+00
5.0e+06
1.0e+07
1.5e+07
2.0e+07
33.5
34.0
34.5
35.0
35.5
Retention Time (min)
#10 TLEDL[+7.017164]LM[+15.994915]GT++
Peptide mix
Top 4 transitions: dotp = 0.89; l:h ppm = 40
Ion
b7+
b6+
y4+
b5+
y7+
Intensity (a.u.)
3e+03
2e+03
1e+03
0e+00
3e+07
6e+07
9e+07
58.0
58.5
59.0
59.5
Retention Time (min)
#11 RTLEDL[+7.017164]LM[+15.994915]GT++
Peptide mix
Top 4 transitions: dotp = 0.98; l:h ppm = 37
Ion
b7+
b8+
b6+
b8++
precursor −64++
Intensity (a.u.)
9e+03
6e+03
3e+03
0e+00
1e+08
2e+08
3e+08
55.5
56.0
56.5
57.0
Retention Time (min)

#12 IRTLEDL[+7.017164]LM[+15.994915]GT++

Peptide mix
Top 4 transitions: dotp = 0.98; l:h ppm = 32

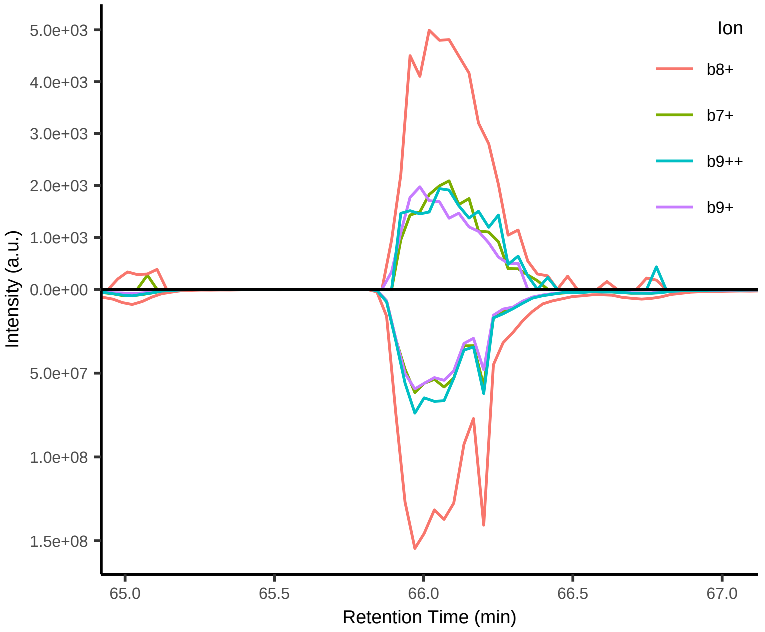

#13 IRTLEDL[+7.017164]LMGT++

Peptide mix
Top 4 transitions: dotp = 0.97; l:h ppm = 28

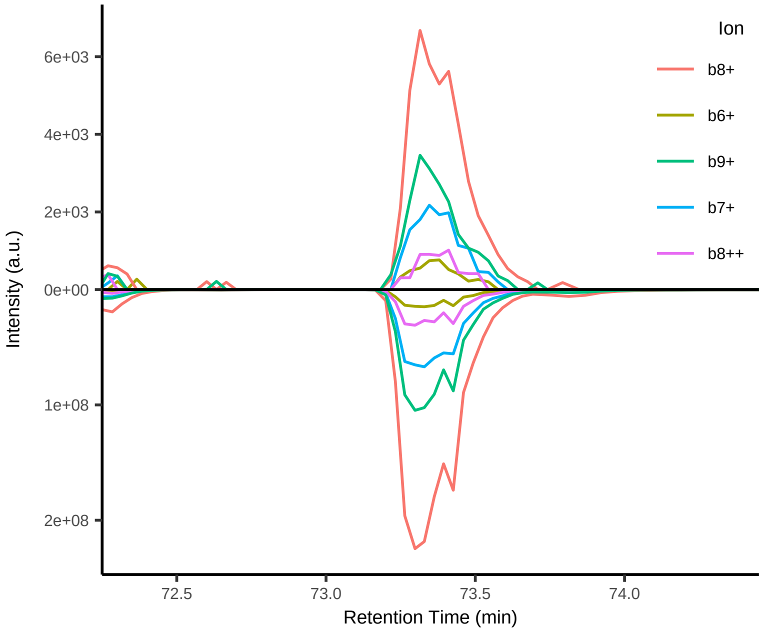

#14 YM[+15.994915]LDL[+7.017164]QPETT++

Peptide mix
Top 4 transitions: dotp = 0.93; l:h ppm = 21

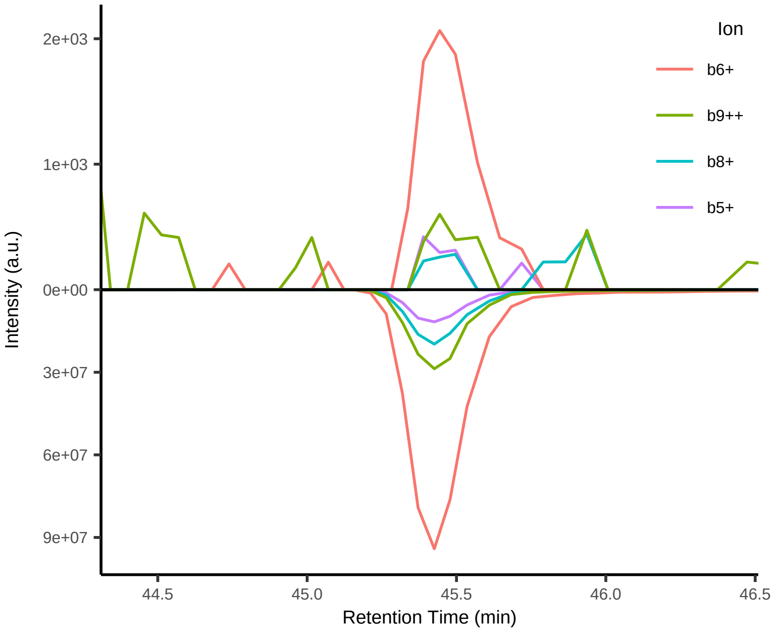

#15 LLMGTLGI[+7.017164]V+

Peptide mix
Top 4 transitions: dotp = 0.91; l:h ppm = 11

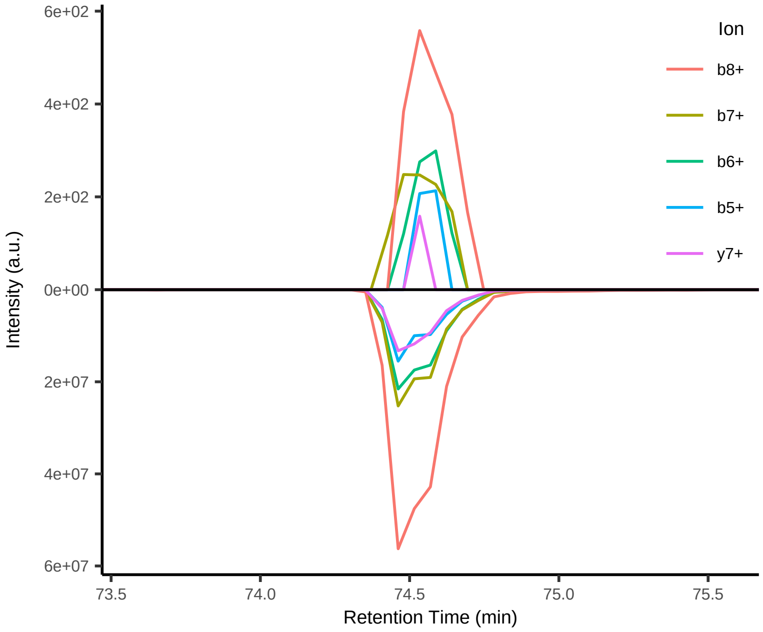

#16 DLLMGTLGI[+7.017164]V++

Peptide mix
Top 4 transitions: dotp = 0.73; l:h ppm = 9

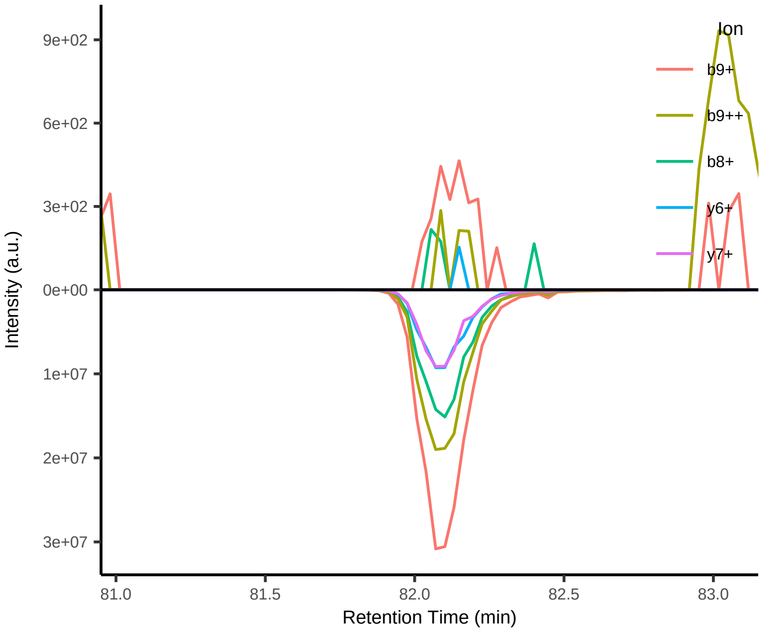

#17 ELQTTI[+7.017164]HDI++

Peptide mix
Top 4 transitions: dotp = 0.72; l:h ppm = 6

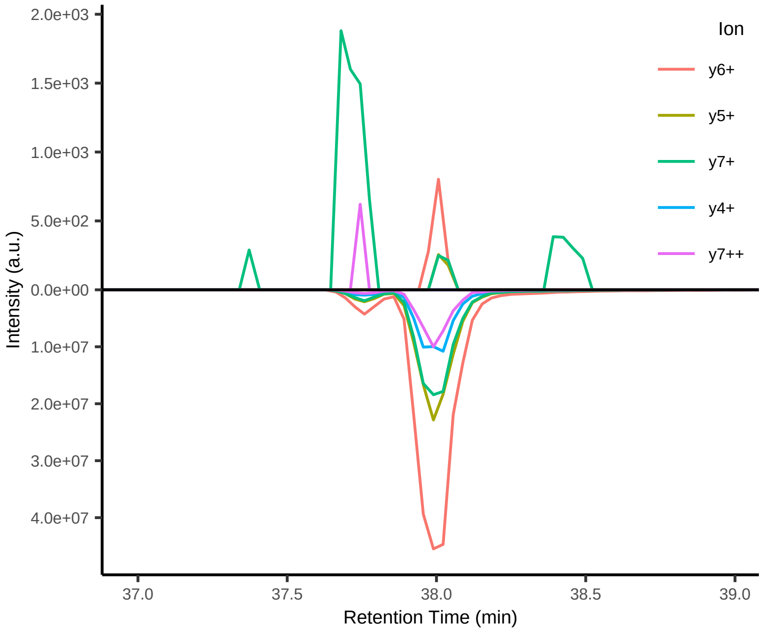

#18 EYML[+7.017164]DL[+7.017164]QPET++

Peptide mix
Top 4 transitions: dotp = NA; l:h ppm = NA

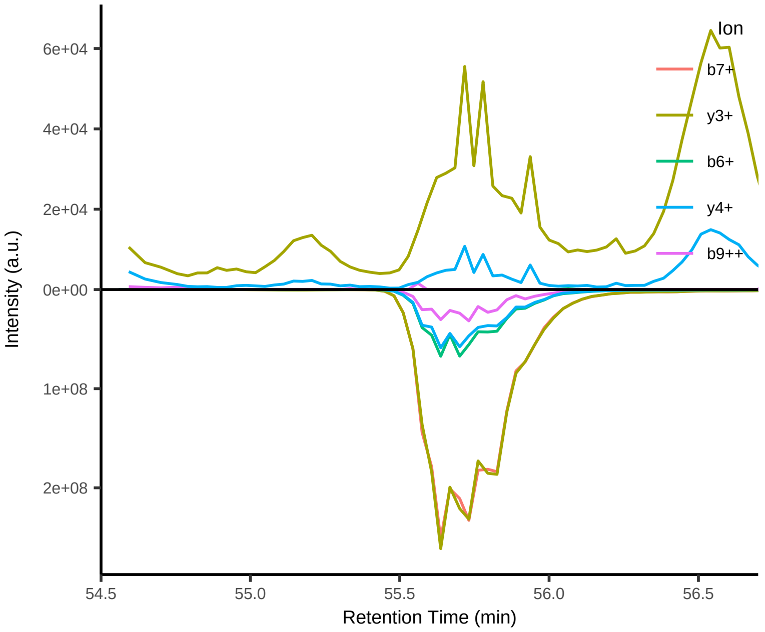

#19 YMLDL[+7.017164]QPETT++

Peptide mix
Top 4 transitions: dotp = NA; l:h ppm = NA

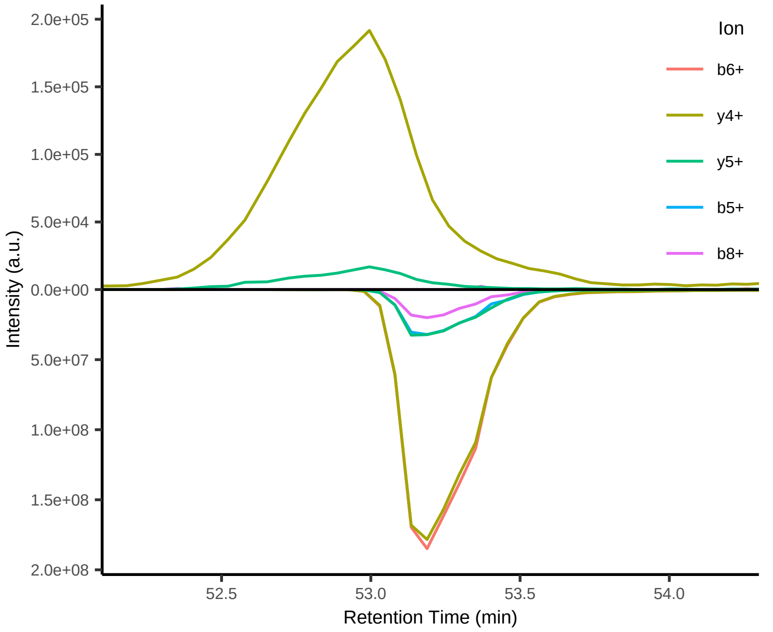

#20 EYM[+15.994915]L[+7.017164]DL[+7.017164]QPET++

Peptide mix
Top 4 transitions: dotp = NA; l:h ppm = NA

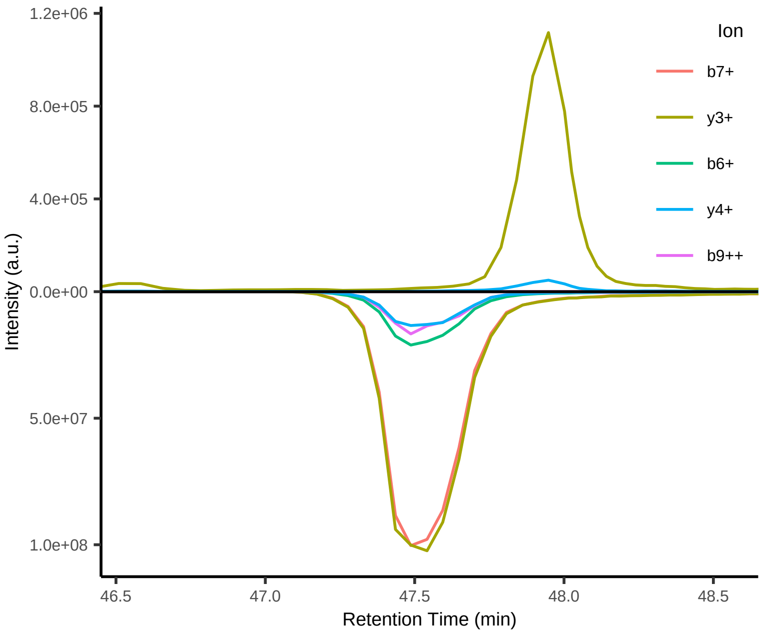

#21 YML[+7.017164]DLQPE+

Peptide mix
Top 4 transitions: dotp = NA; l:h ppm = NA

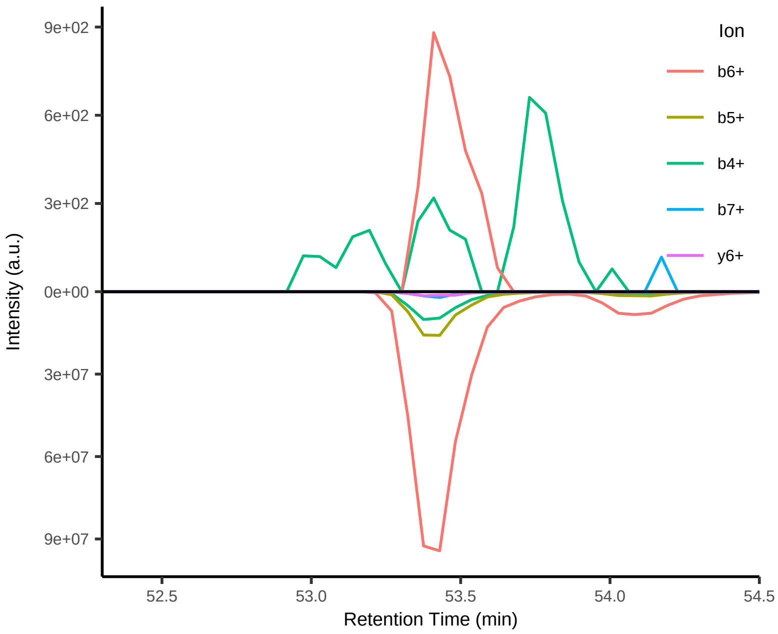

#22 YM[+15.994915]LDL[+7.017164]QPET++

Peptide mix
Top 4 transitions: dotp = NA; l:h ppm = NA

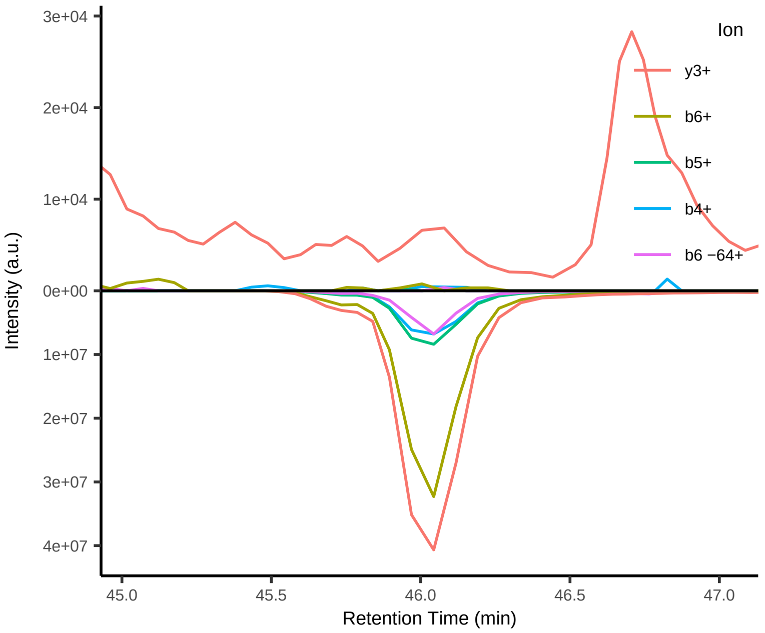

#23 YMLDL[+7.017164]QPET++

Peptide mix
Top 4 transitions: dotp = NA; l:h ppm = NA

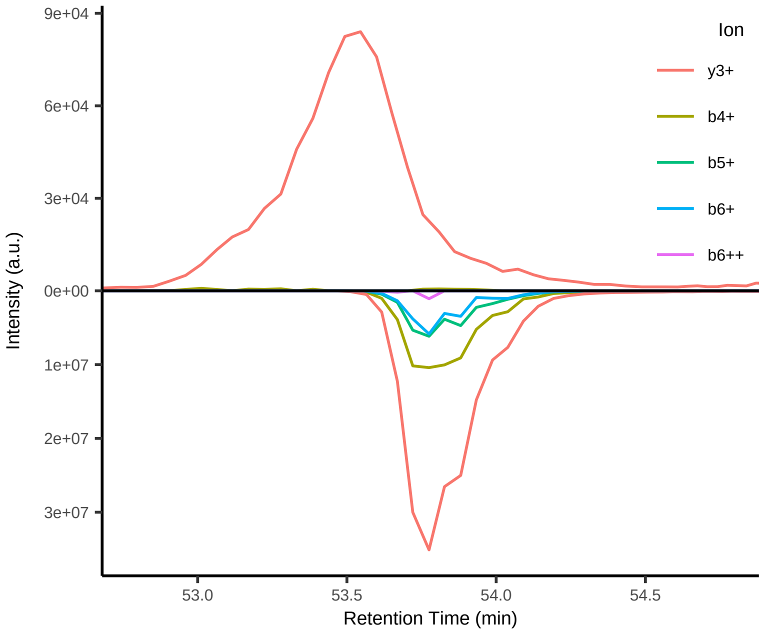

#24 MLDL[+7.017164]QPET++

Peptide mix
Top 4 transitions: dotp = NA; l:h ppm = NA

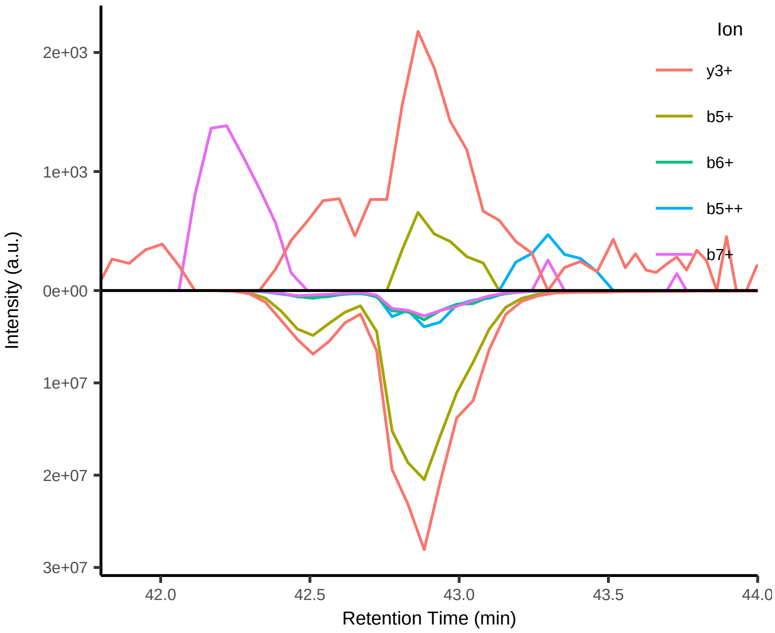

#25 LLM[+15.994915]GTLGI[+7.017164]V++

Peptide mix
Top 4 transitions: dotp = NA; l:h ppm = NA

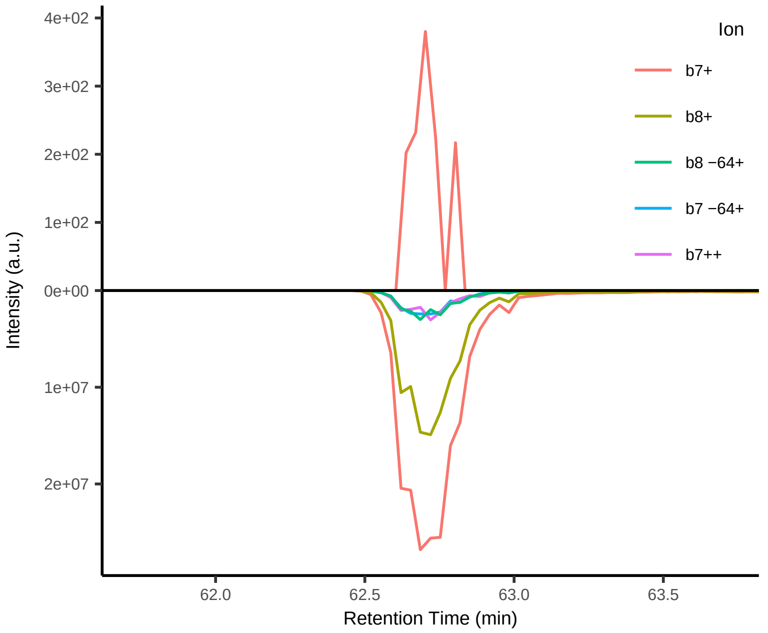

#26 EDLLMGTLGI[+7.017164]V++

Peptide mix
Top 4 transitions: dotp = NA; l:h ppm = NA

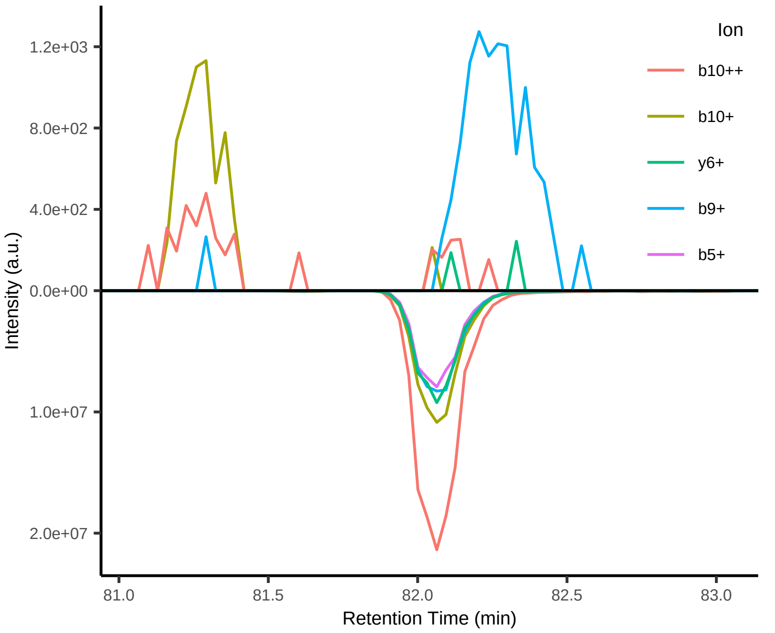

#27 DLLM[+15.994915]GTLGI[+7.017164]V++

Peptide mix
Top 4 transitions: dotp = NA; l:h ppm = NA

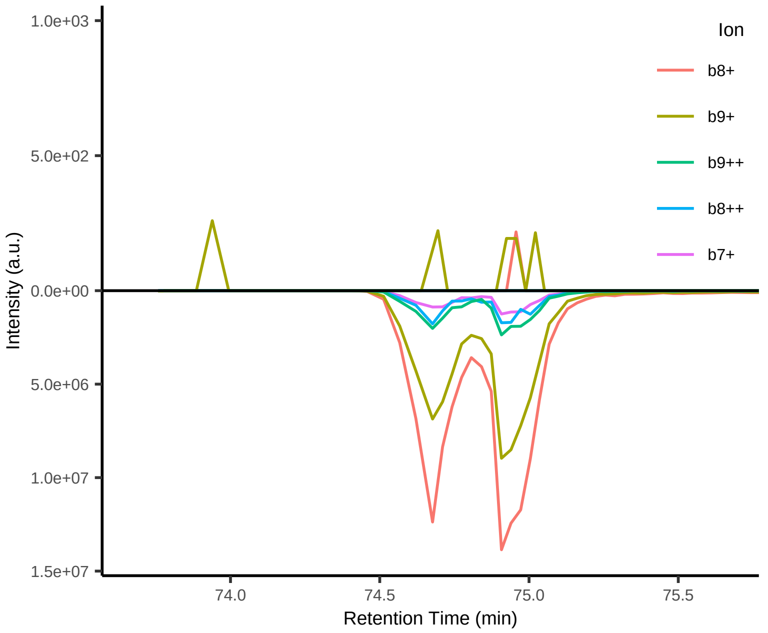

#28 MLDL[+7.017164]QPETT+

Peptide mix
Top 4 transitions: dotp = NA; l:h ppm = NA

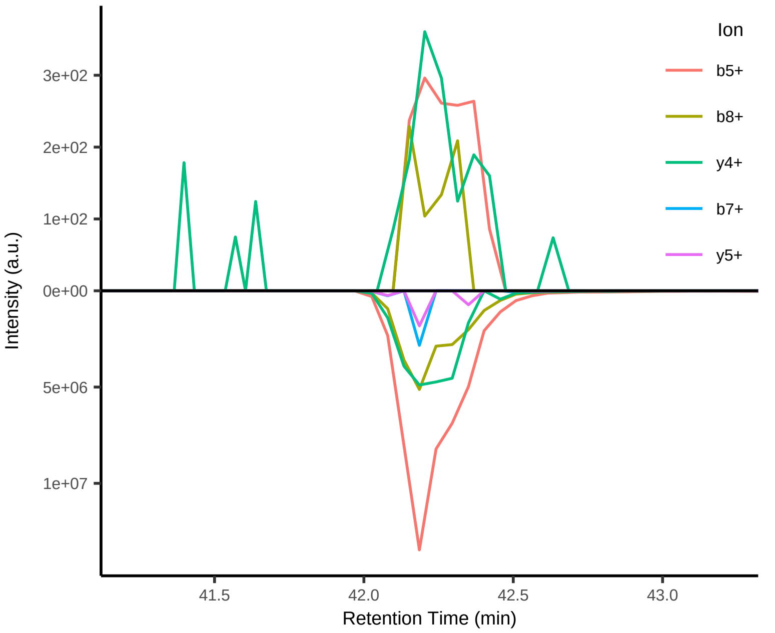

#29 EDLLM[+15.994915]GTLGI[+7.017164]V++

Peptide mix
Top 4 transitions: dotp = NA; l:h ppm = NA

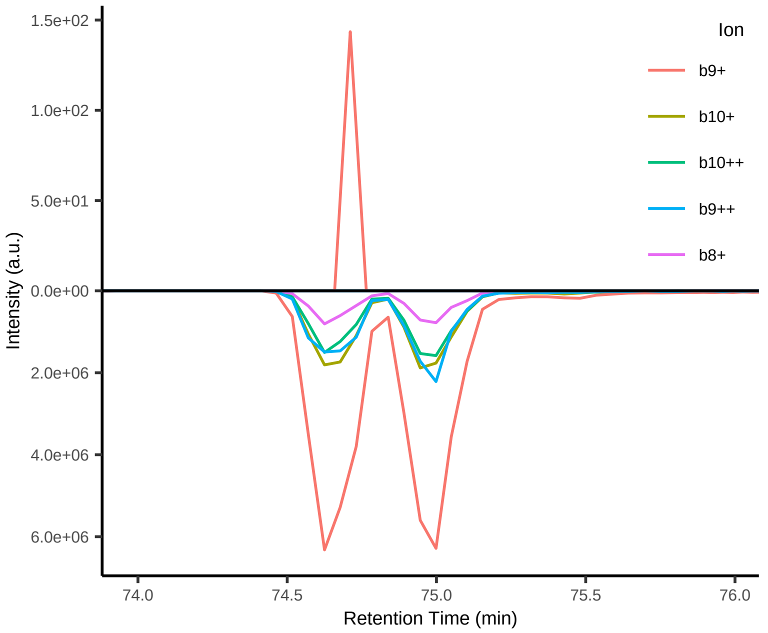

#30 M[+15.994915]LDL[+7.017164]QPET++

Peptide mix
Top 4 transitions: dotp = NA; l:h ppm = NA

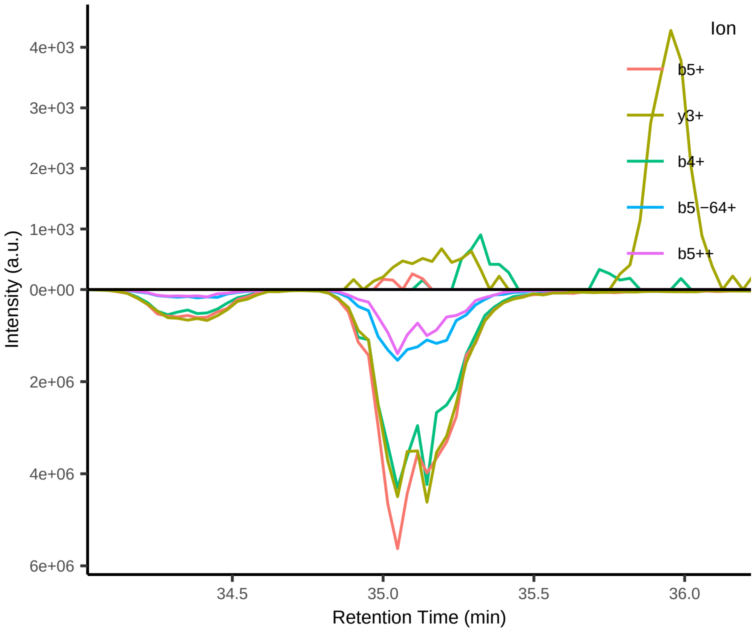

#31 YM[+15.994915]L[+7.017164]DLQPE++

Peptide mix
Top 4 transitions: dotp = NA; l:h ppm = NA

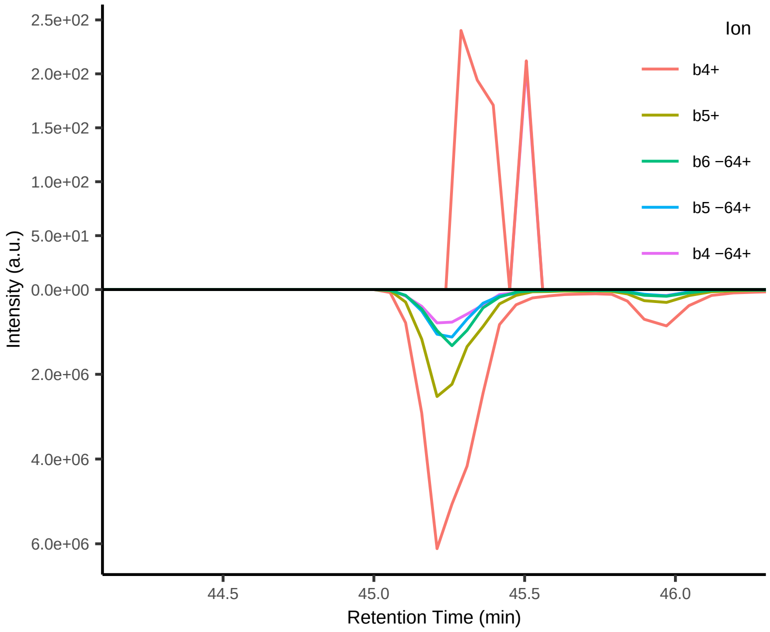

#32 TLHEYML[+7.017164]DLQP++

Peptide mix
Top 4 transitions: dotp = NA; l:h ppm = NA

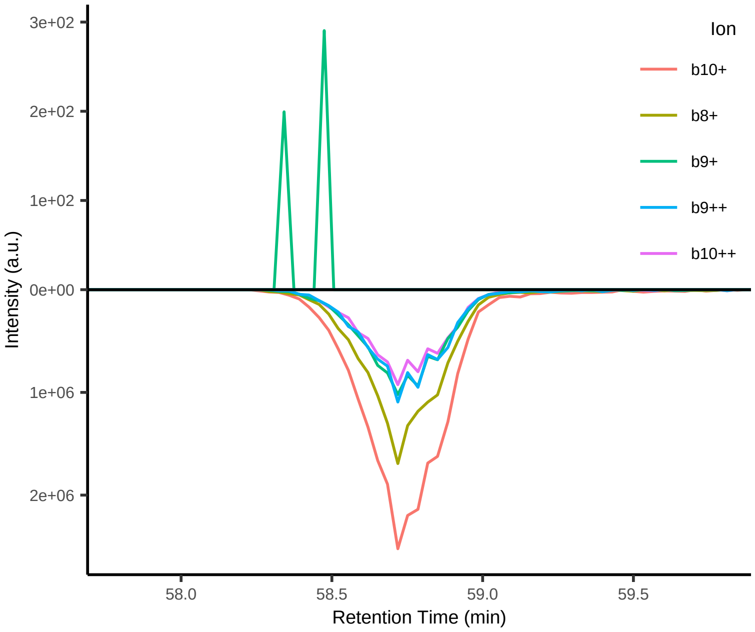

#33 TLHEYM[+15.994915]L[+7.017164]DLQP++

Peptide mix
Top 4 transitions: dotp = NA; l:h ppm = NA

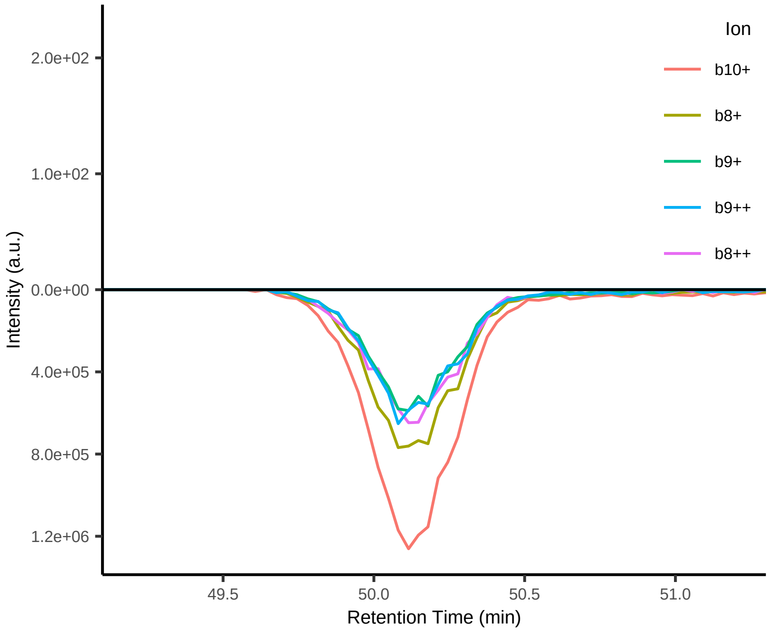

#34 AAL[+7.017164]ENTHLL++

Peptide mix
Top 4 transitions: dotp = NA; l:h ppm = NA

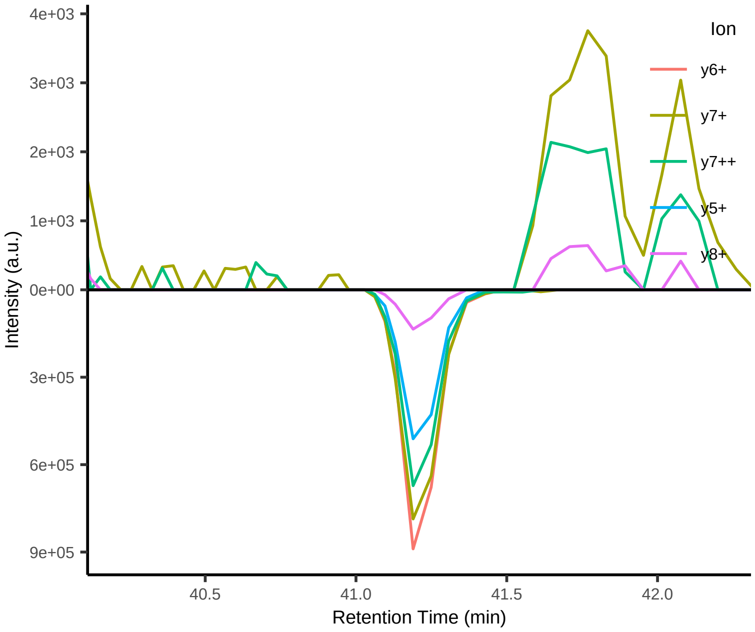

#35 TL[+7.017164]HEYMLDL++

Peptide mix
Top 4 transitions: dotp = NA; l:h ppm = NA

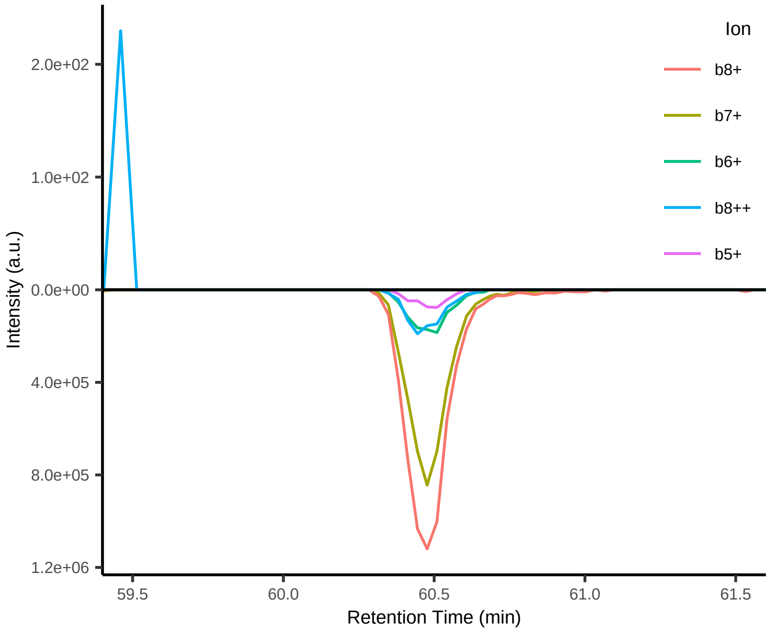

#36 FGPV[+6.013809]NHEEL++

Peptide mix
Top 4 transitions: dotp = NA; l:h ppm = NA

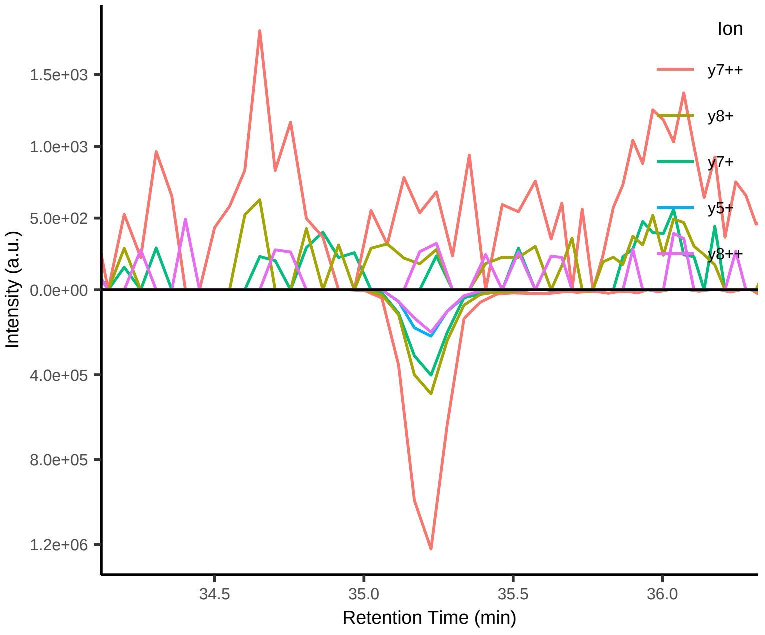

#37 AL[+7.017164]NEKLVNL++

Peptide mix
Top 4 transitions: dotp = NA; l:h ppm = NA

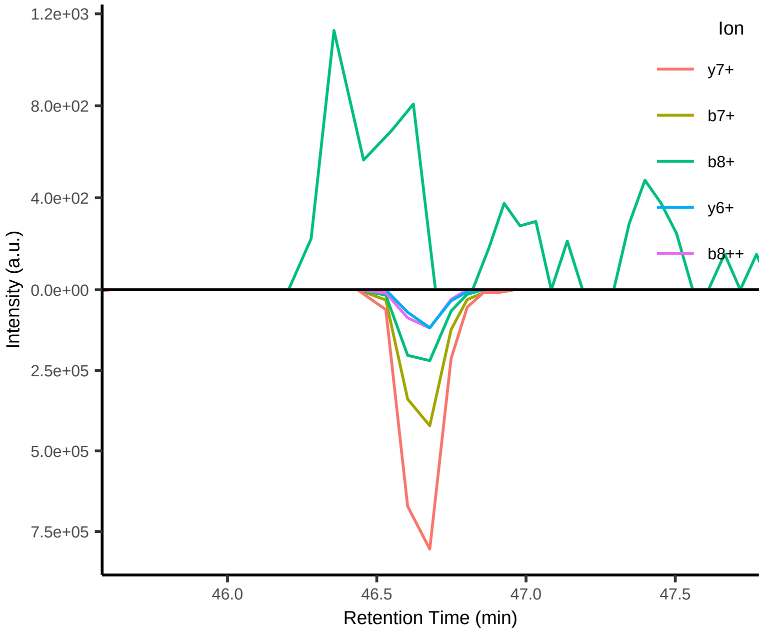

#38 KAL[+7.017164]INADEL++

Peptide mix
Top 4 transitions: dotp = NA; l:h ppm = NA

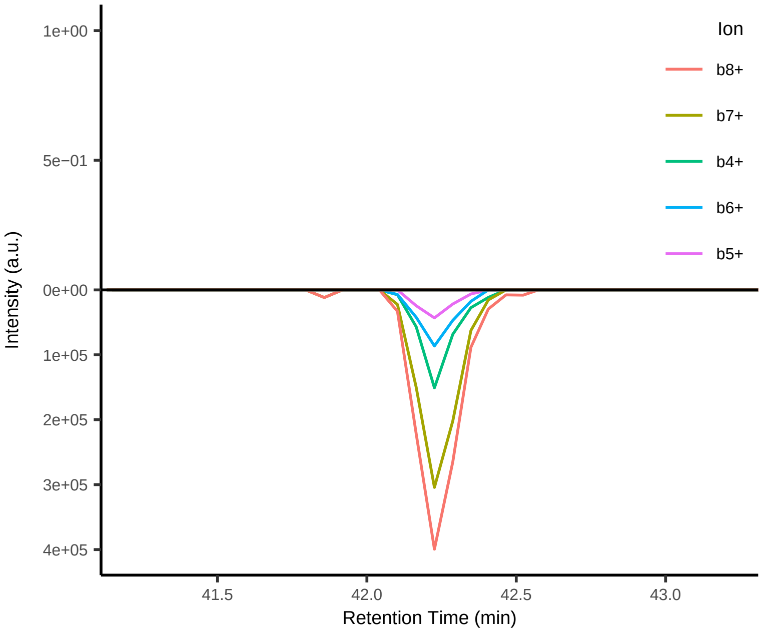

#39 TL[+7.017164]HEYM[+15.994915]LDL++

Peptide mix
Top 4 transitions: dotp = NA; l:h ppm = NA

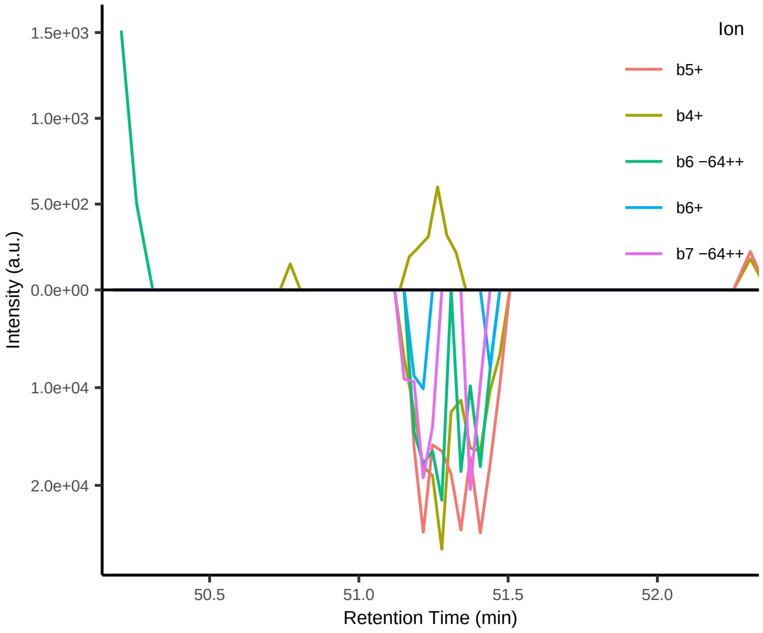

#40 TASEFDSAIAQDK[+8.014199]++

Peptide mix
Top 4 transitions: dotp = 0.99; l:h ppm = 44962

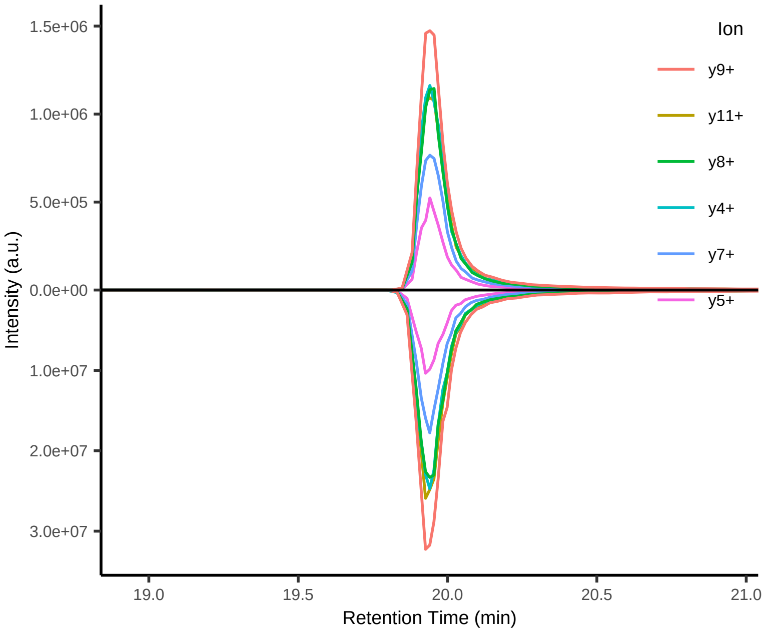

#41 DIPVPKPK[+8.014199]++

Peptide mix
Top 4 transitions: dotp = 0.99; l:h ppm = 835

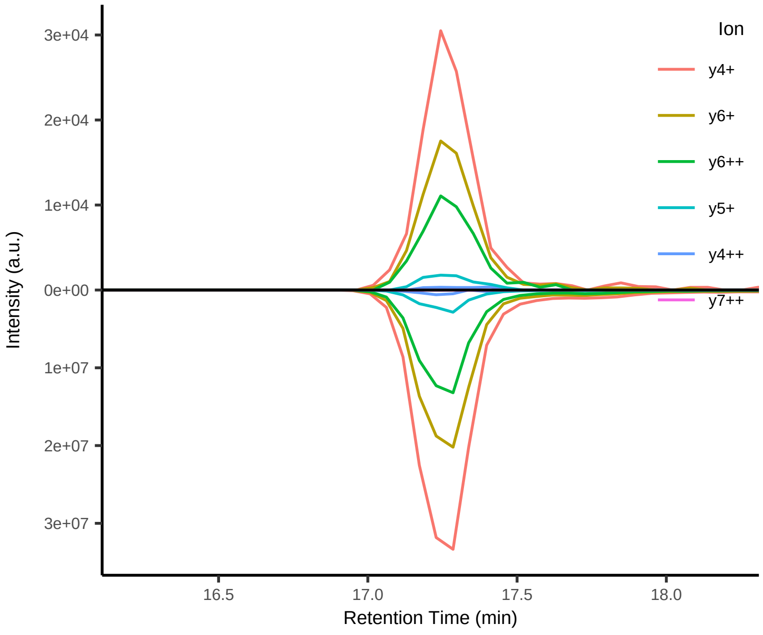

#42 ELGQSGVDTYLQTK[+8.014199]++

Peptide mix
Top 4 transitions: dotp = 0.97; l:h ppm = 285

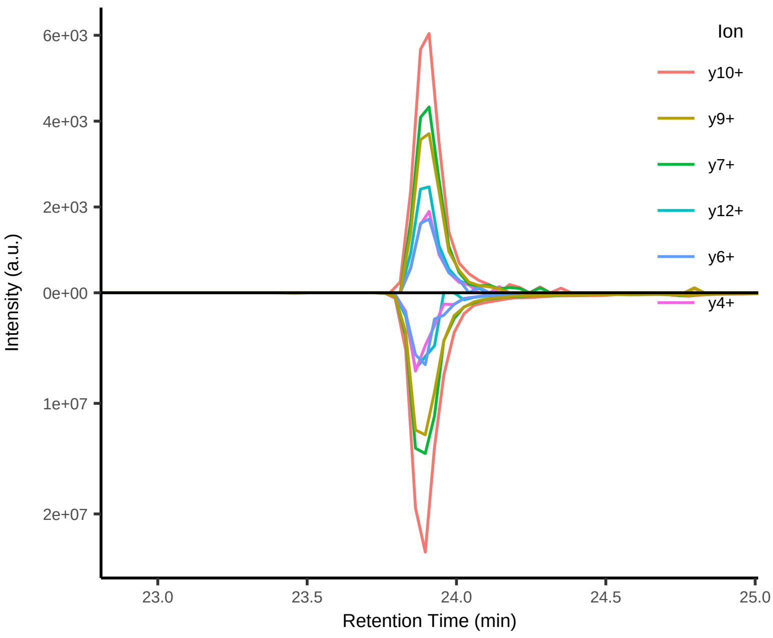

#43 GISNEGQNASIK[+8.014199]++

Peptide mix
Top 4 transitions: dotp = 0.98; l:h ppm = 221

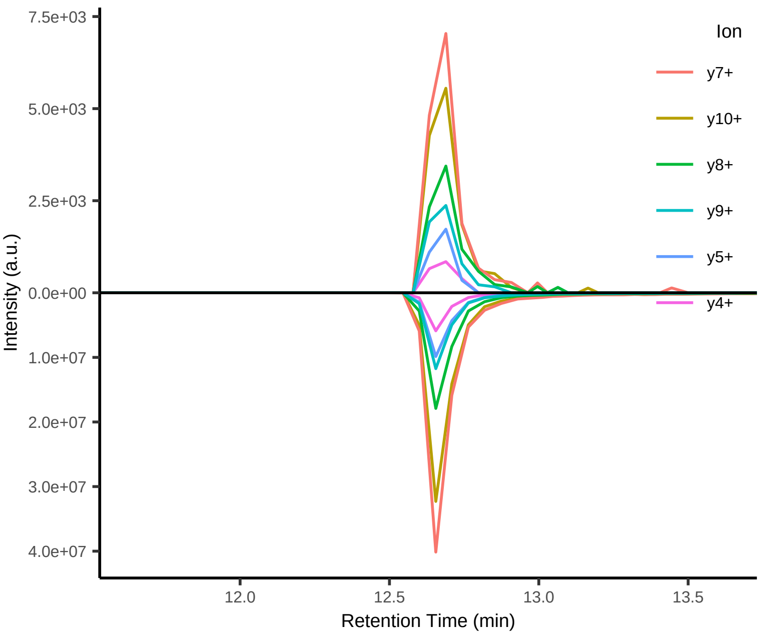

#44 NGFILDGFPR[+10.008269]++

Peptide mix
Top 4 transitions: dotp = 0.97; l:h ppm = 202

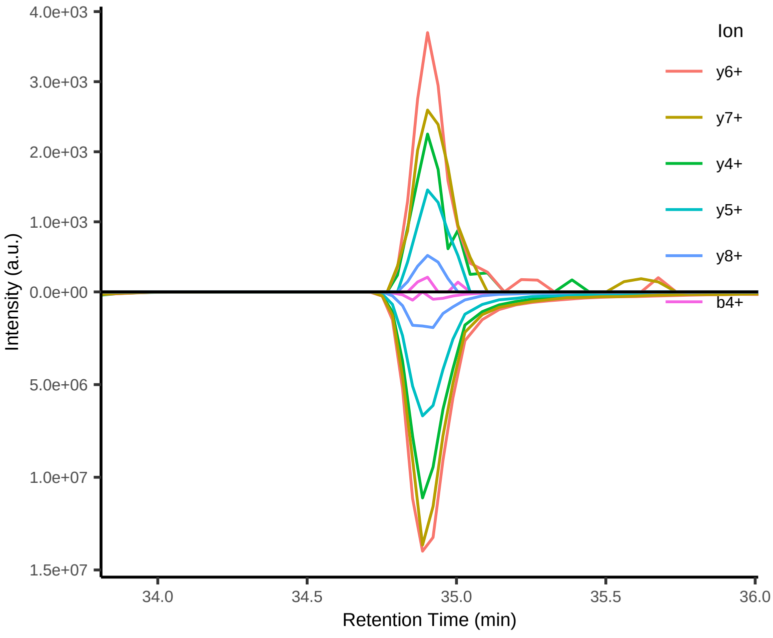

#45 HVLTSIGEK[+8.014199]++

Peptide mix
Top 4 transitions: dotp = 0.98; l:h ppm = 173

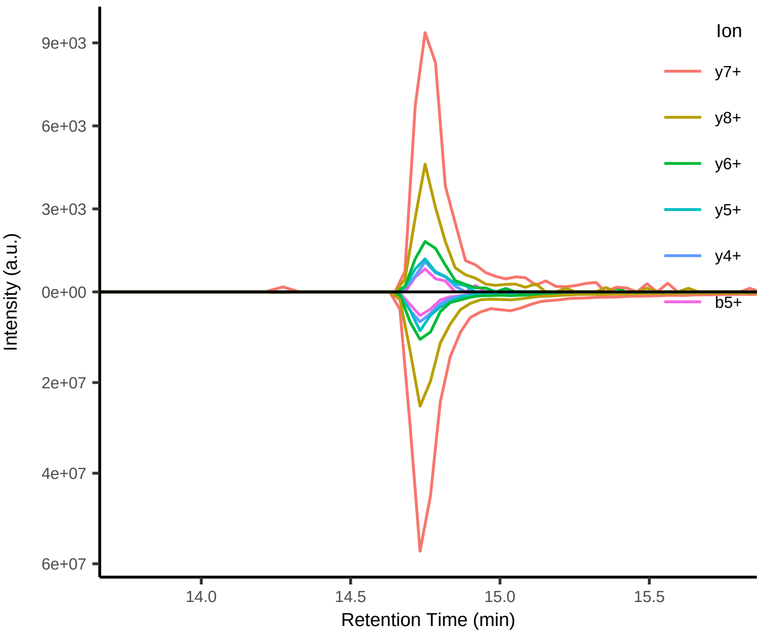

#46 IGDYAGIK[+8.014199]++

Peptide mix
Top 4 transitions: dotp = 0.98; l:h ppm = 150

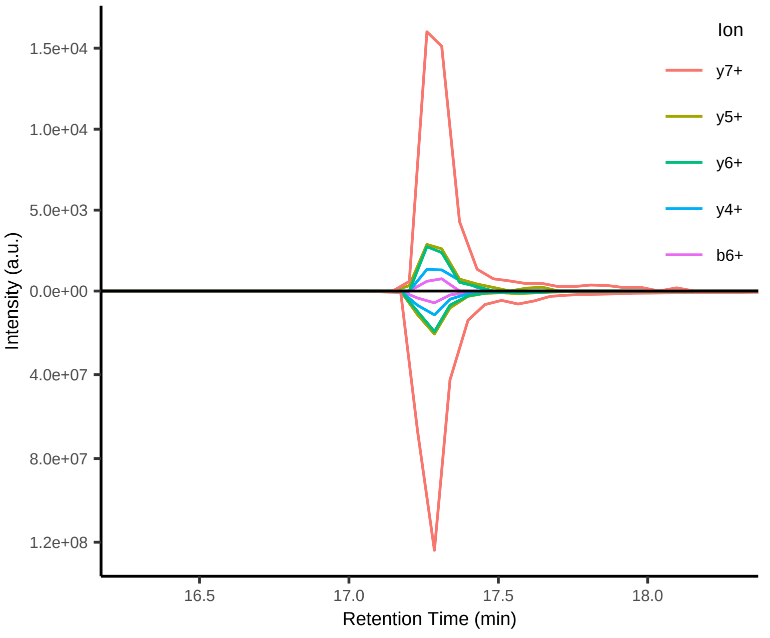

#47 SFANQPLEVVYSK[+8.014199]++

Peptide mix
Top 4 transitions: dotp = 0.94; l:h ppm = 145

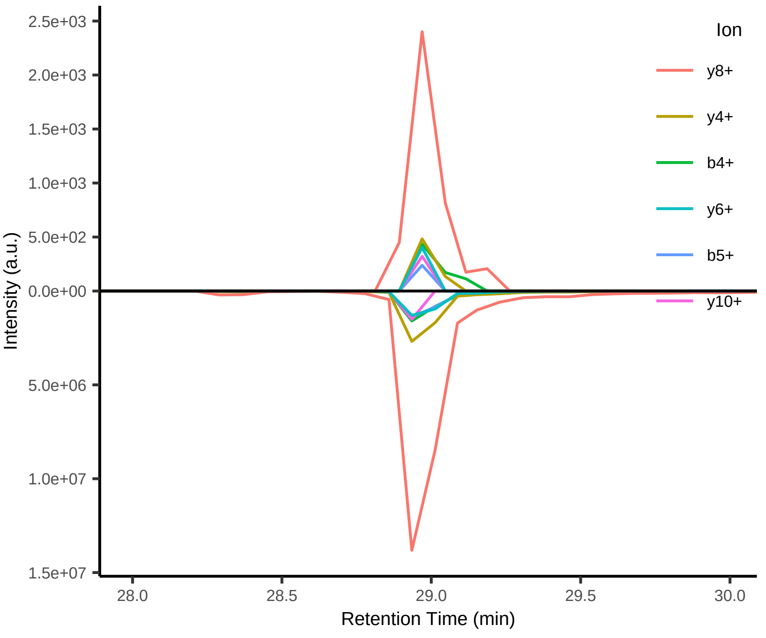

#48 LSSEAPALFQFDLK[+8.014199]++

Peptide mix
Top 4 transitions: dotp = 0.54; l:h ppm = 142

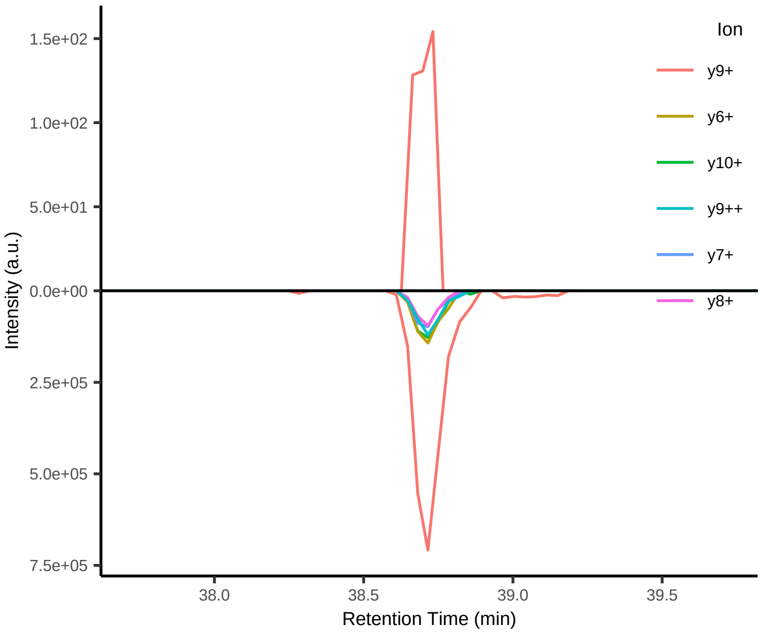

#49 SAAGAFGPELSR[+10.008269]++

Peptide mix
Top 4 transitions: dotp = 0.99; l:h ppm = 106

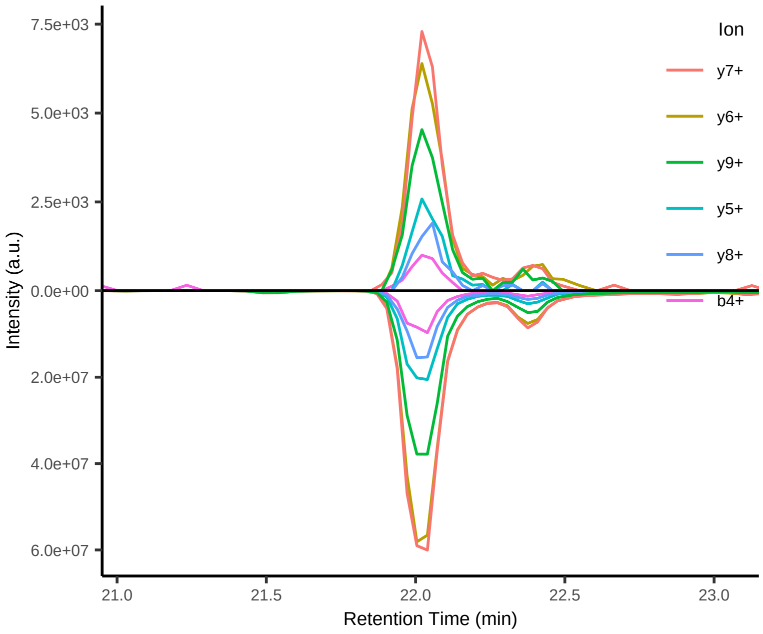

#50 ELASGLSFPVGFK[+8.014199]++

Peptide mix
Top 4 transitions: dotp = 0.80; l:h ppm = 104

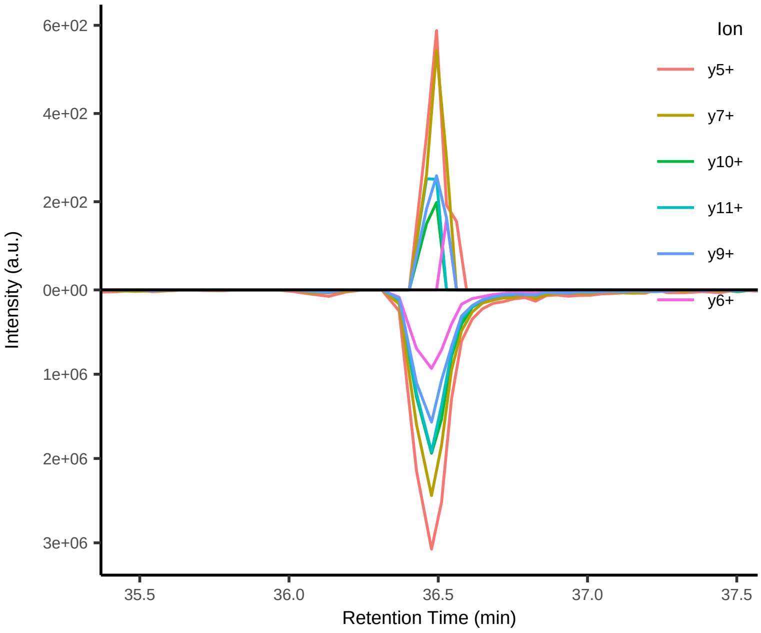

#51 GLILVGGYGTR[+10.008269]++

Peptide mix
Top 4 transitions: dotp = 0.86; l:h ppm = 95

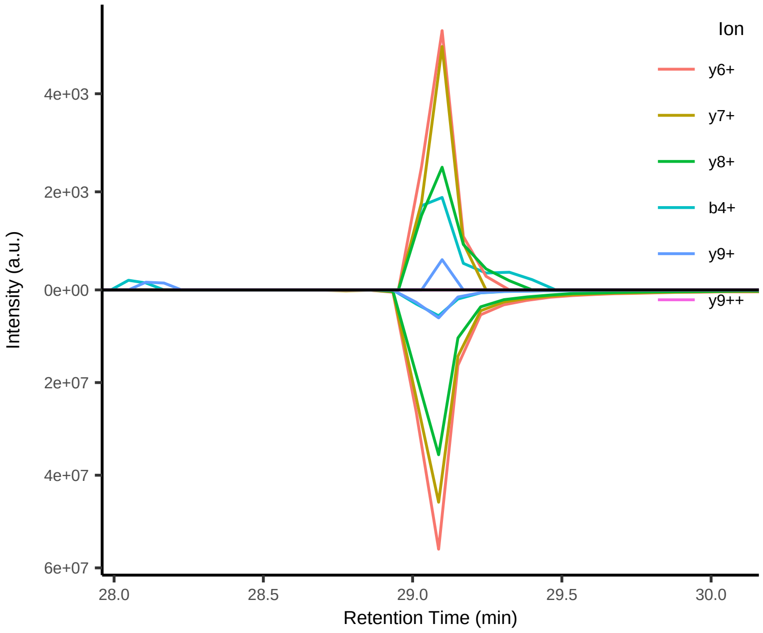

#52 GILFVGSGVSGGEEGAR[+10.008269]++

Peptide mix
Top 4 transitions: dotp = 0.95; l:h ppm = 89

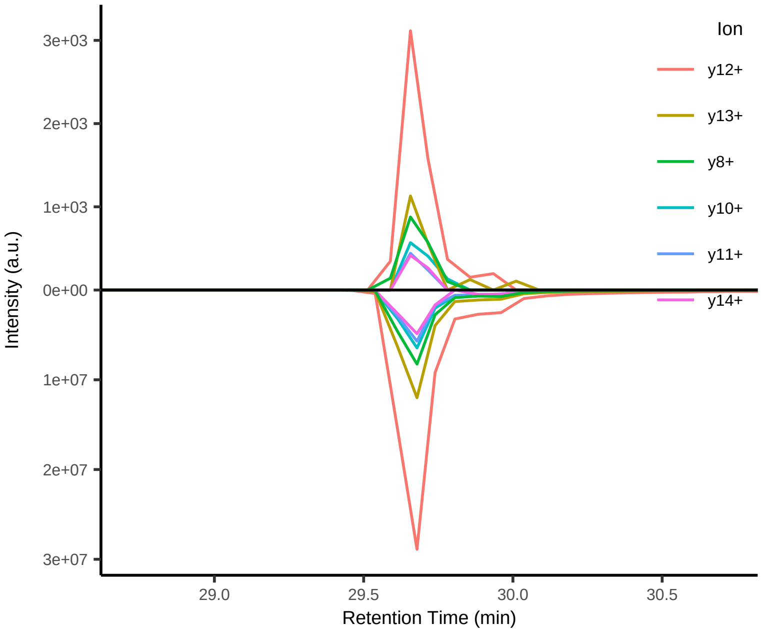

#53 LTILEELR[+10.008269]++

Peptide mix
Top 4 transitions: dotp = 0.90; l:h ppm = 25

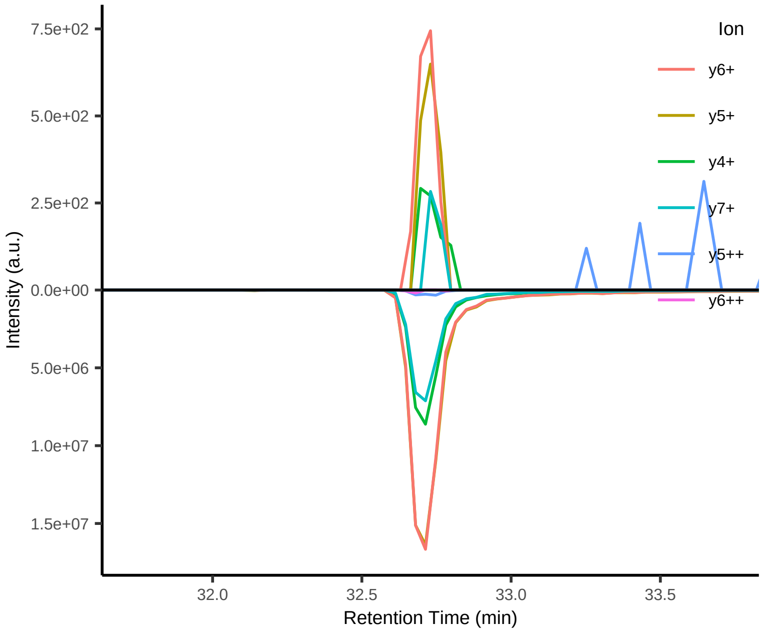

#54 SSAAPPPPPR[+10.008269]++

Peptide mix
Top 4 transitions: dotp = 0.93; l:h ppm = 25

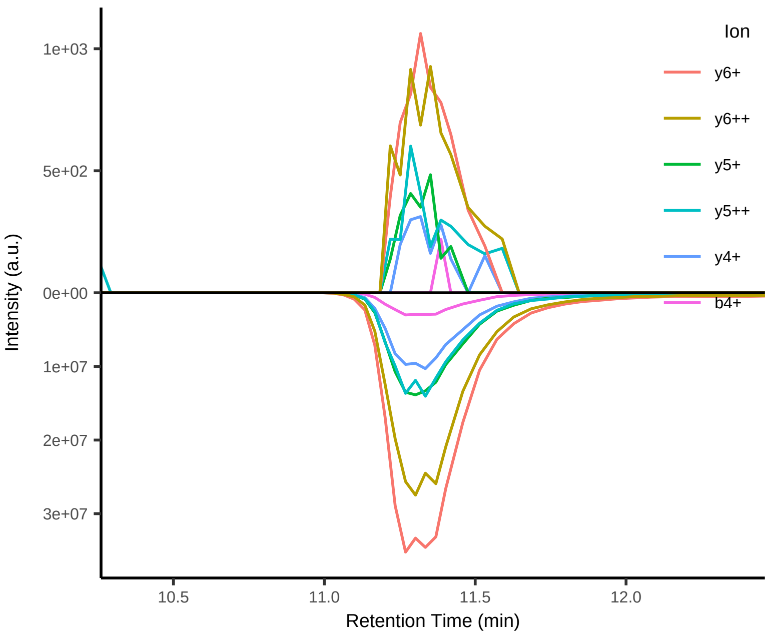

#55 IAESL[+7.017164]PVV+

Peptide mix
Top 4 transitions: dotp = 0.86; l:h ppm = 611

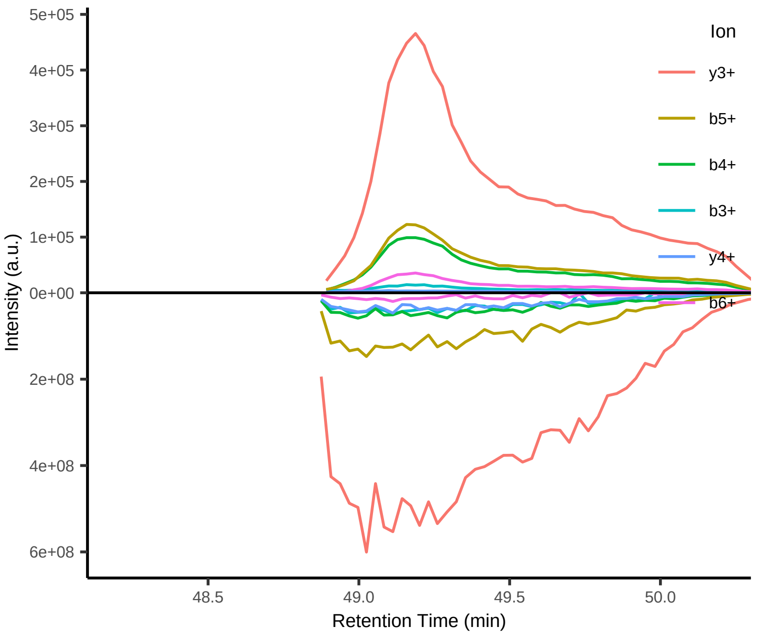

#56 SRFTGATII[+7.017164]++

Peptide mix
Top 4 transitions: dotp = 0.96; l:h ppm = 538

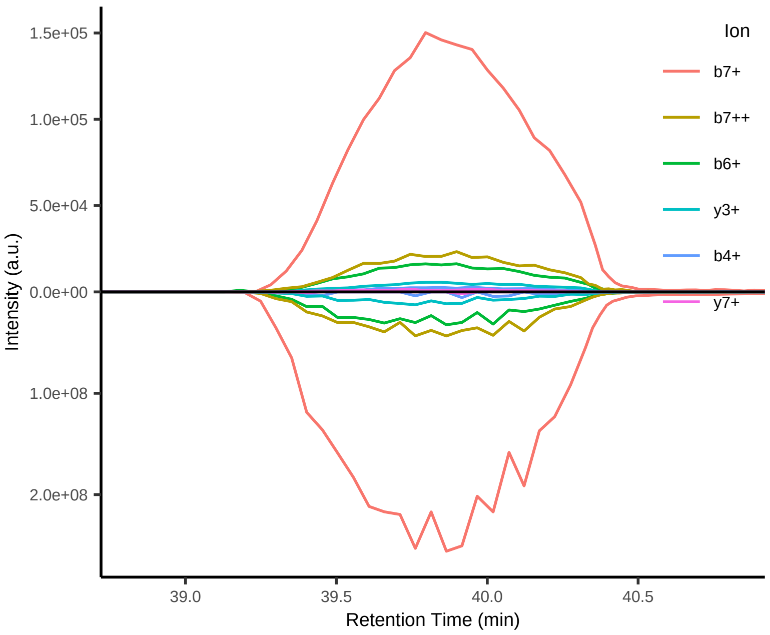

#57 KSTL[+7.017164]VRLLF++

Peptide mix
Top 4 transitions: dotp = 0.99; l:h ppm = 475

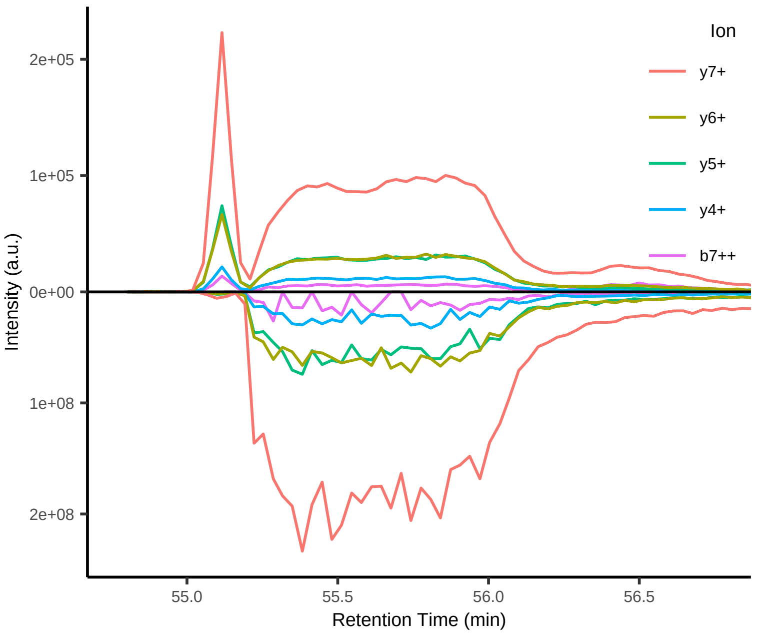

#58 L[+7.017164]NPNDPYHTYY++

Peptide mix
Top 4 transitions: dotp = 0.97; l:h ppm = 405

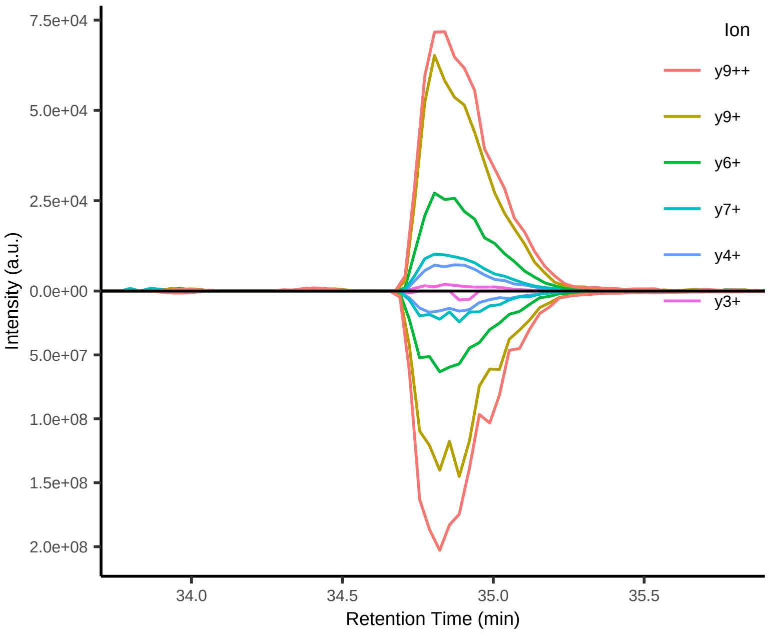

#59 HTYYRHK[+8.014199]V++

Peptide mix
Top 4 transitions: dotp = 0.96; l:h ppm = 381

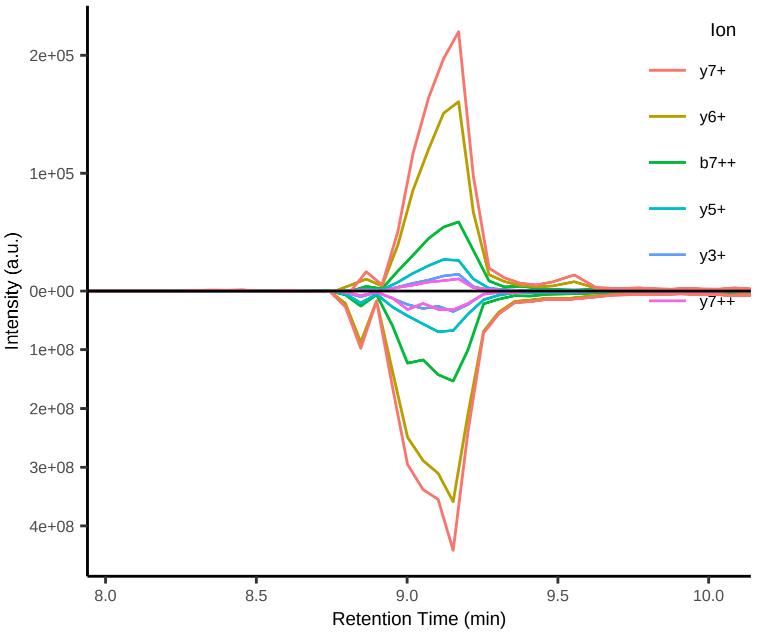

#60 SRL[+7.017164]SKEELI++

Peptide mix
Top 4 transitions: dotp = 0.99; l:h ppm = 331

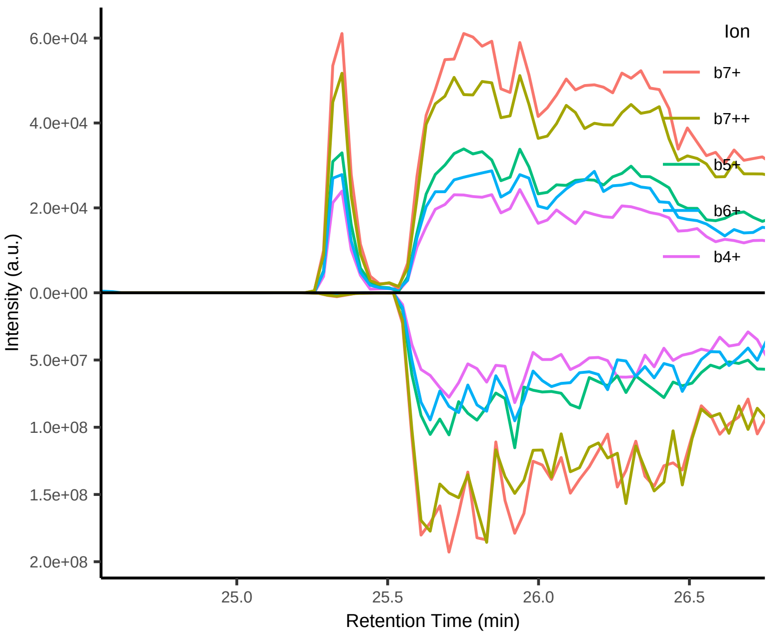

#61 M[+15.994915]LWLPHWGL[+7.017164]++

Peptide mix
Top 4 transitions: dotp = 0.94; l:h ppm = 310

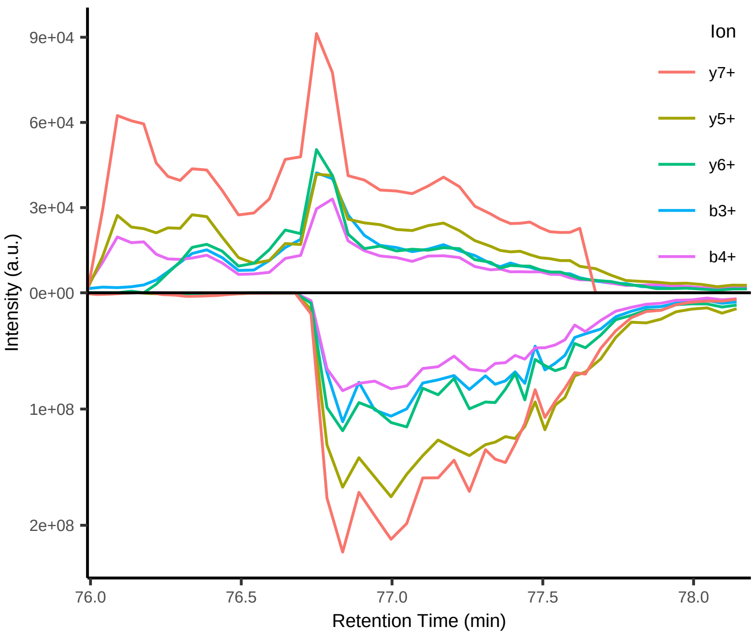

#62 MLWLPHWGL[+7.017164]++

Peptide mix
Top 4 transitions: dotp = 0.96; l:h ppm = 296

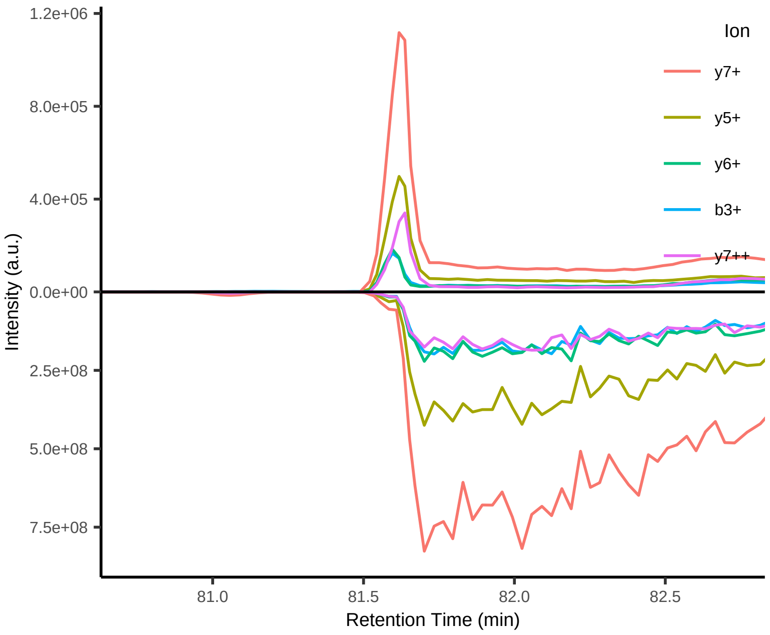

#63 RVRKELQEL[+7.017164]+++

Peptide mix
Top 4 transitions: dotp = 0.56; l:h ppm = 239

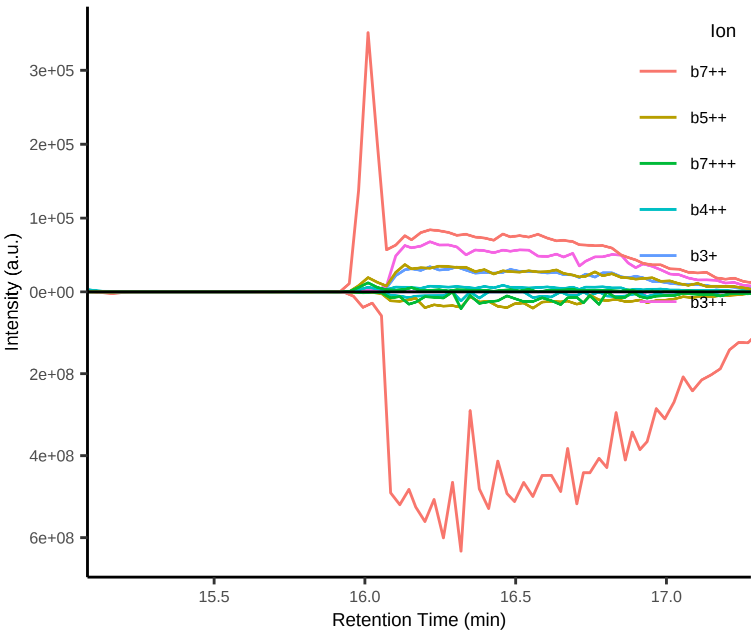

#64 KVAKGHMK[+8.014199]L++

Peptide mix
Top 4 transitions: dotp = 0.95; l:h ppm = 235

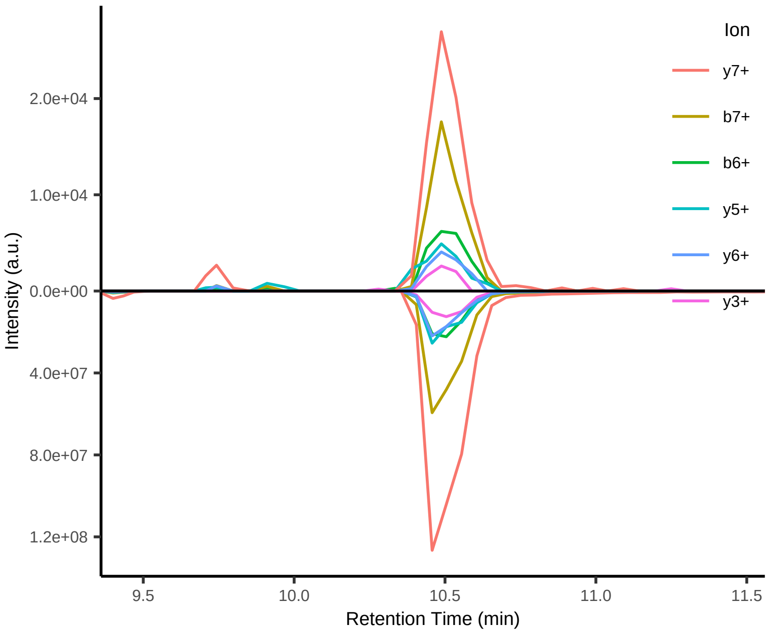

#65 FLM[+15.994915]LWL[+7.017164]PHW++

Peptide mix
Top 4 transitions: dotp = 0.87; l:h ppm = 208

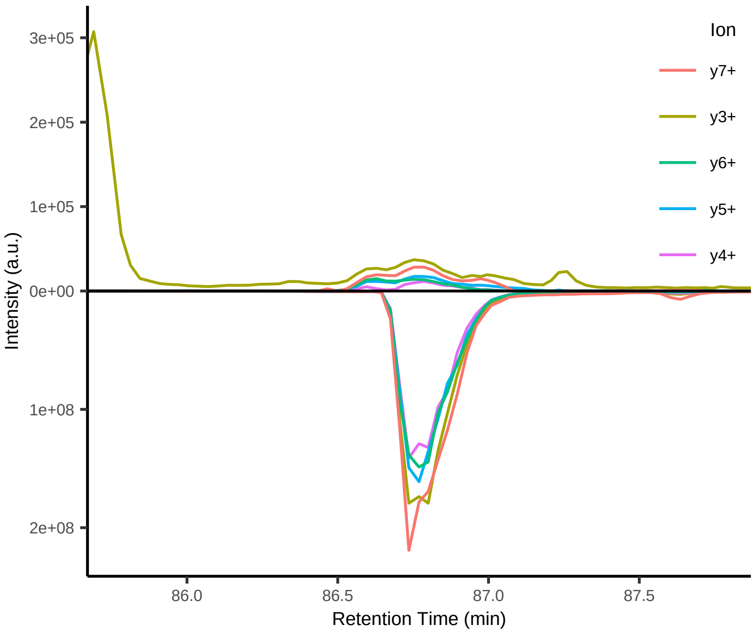

#66 YHTYYRHK[+8.014199]V++

Peptide mix
Top 4 transitions: dotp = 0.98; l:h ppm = 196

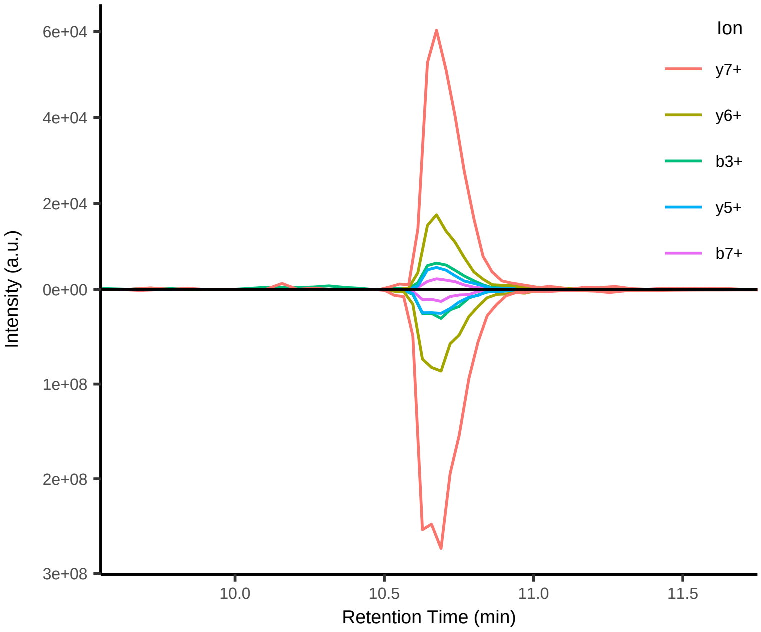

#67 GKSTL[+7.017164]VRLLF+++

Peptide mix
Top 4 transitions: dotp = 0.98; l:h ppm = 189

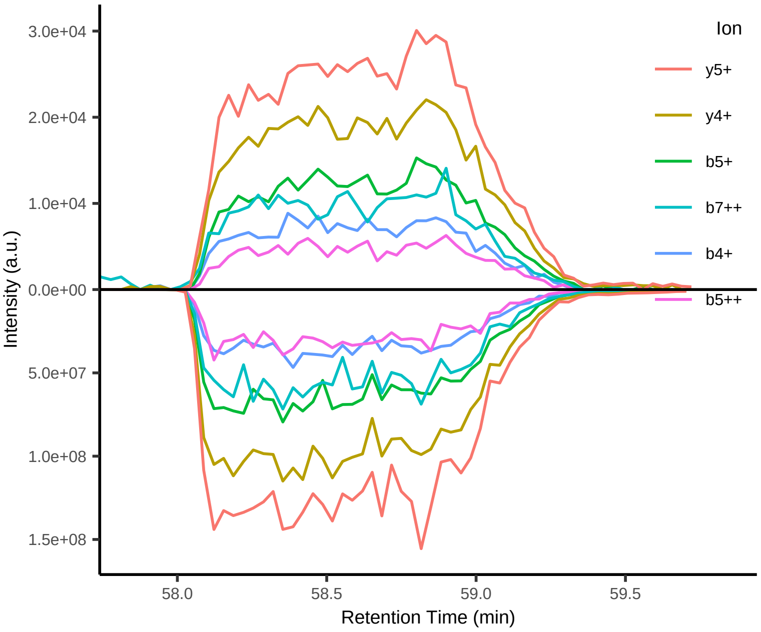

#68 RPMV[+6.013809]RSFTM[+15.994915]+++

Peptide mix
Top 4 transitions: dotp = 0.74; l:h ppm = 188

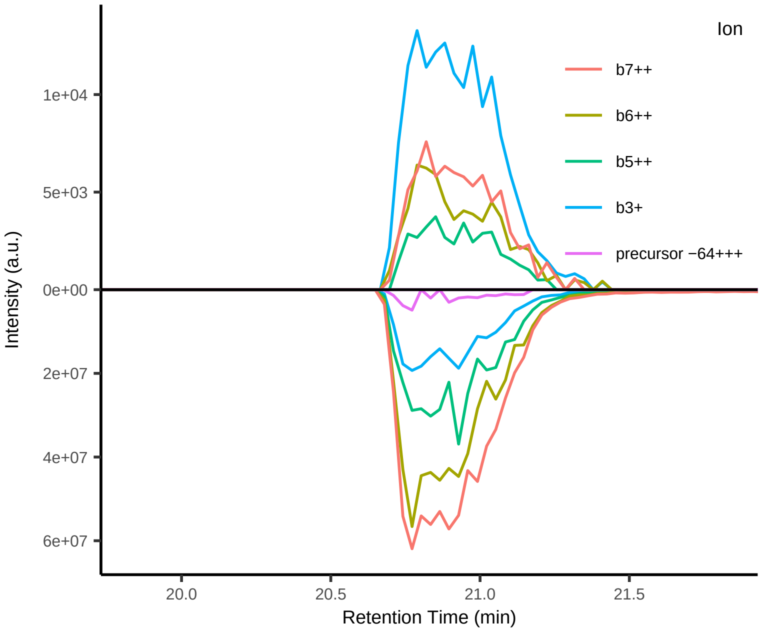

#69 KVAKGHM[+15.994915]K[+8.014199]L+++

Peptide mix
Top 4 transitions: dotp = 0.96; l:h ppm = 178

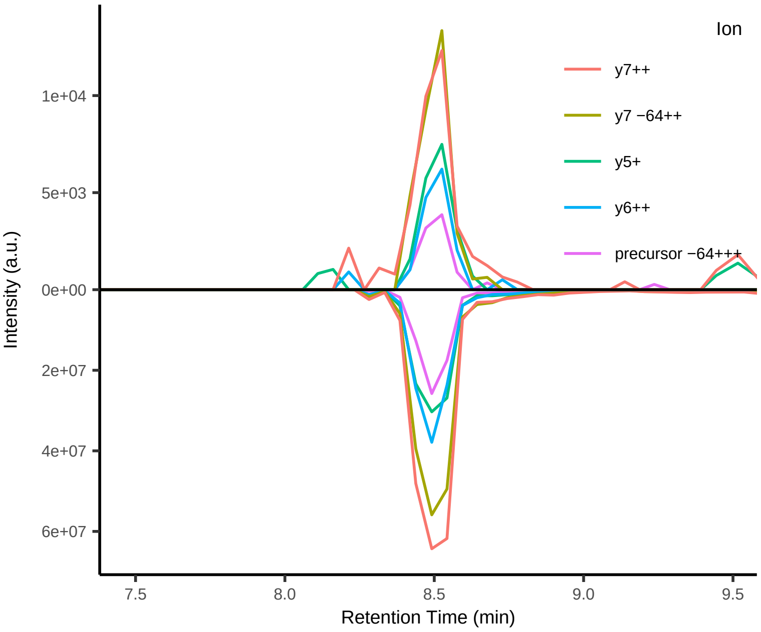

#70 KSTL[+7.017164]VRLL++

Peptide mix
Top 4 transitions: dotp = 0.96; l:h ppm = 178

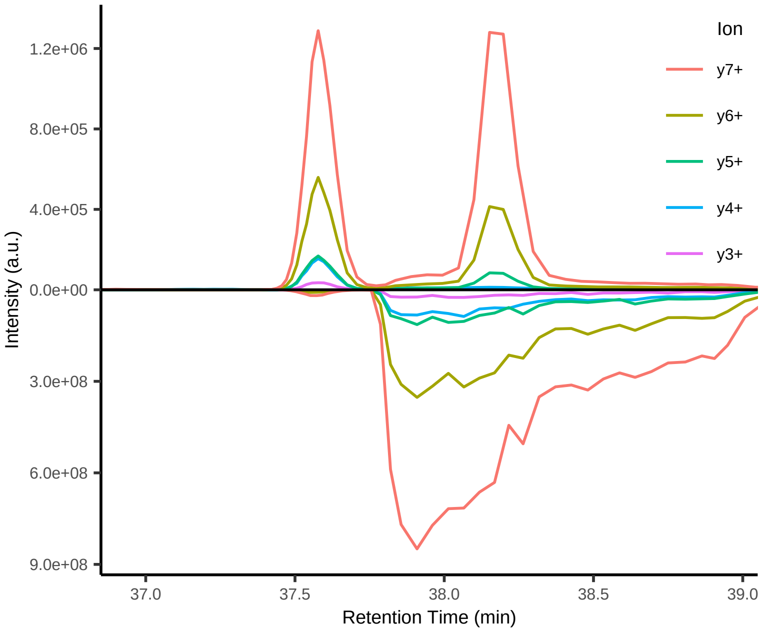

#71 RPMV[+6.013809]RSFTM+++

Peptide mix
Top 4 transitions: dotp = 0.82; l:h ppm = 177

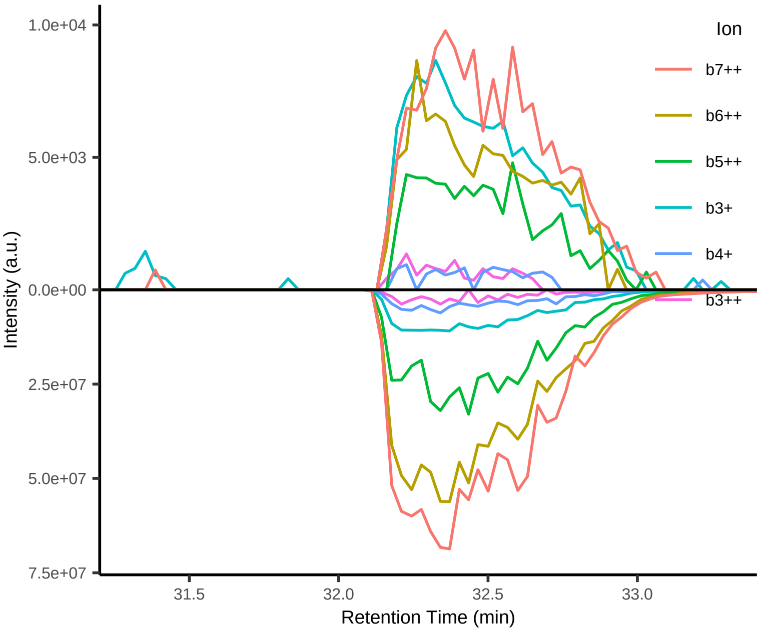

#72 MATELGIVL[+7.017164]+

Peptide mix
Top 4 transitions: dotp = 0.86; l:h ppm = 174

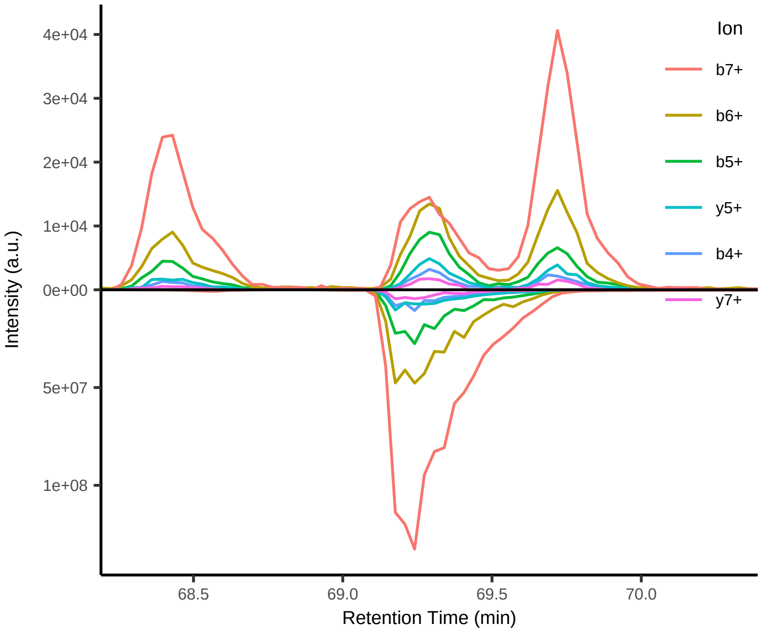

#73 FGTTV[+6.013809]PFTSW++

Peptide mix
Top 4 transitions: dotp = 0.92; l:h ppm = 170

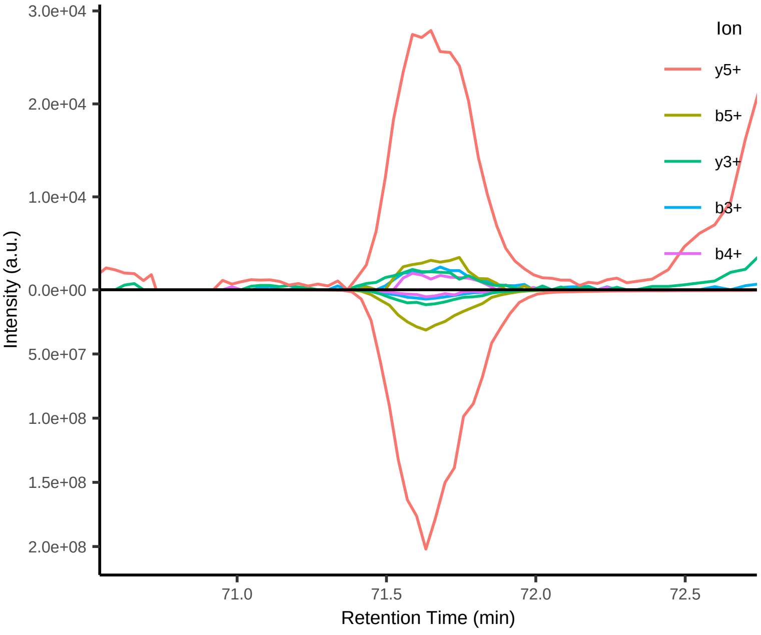

#74 FL[+7.017164]NPNDPYHTY++

Peptide mix
Top 4 transitions: dotp = 0.98; l:h ppm = 165

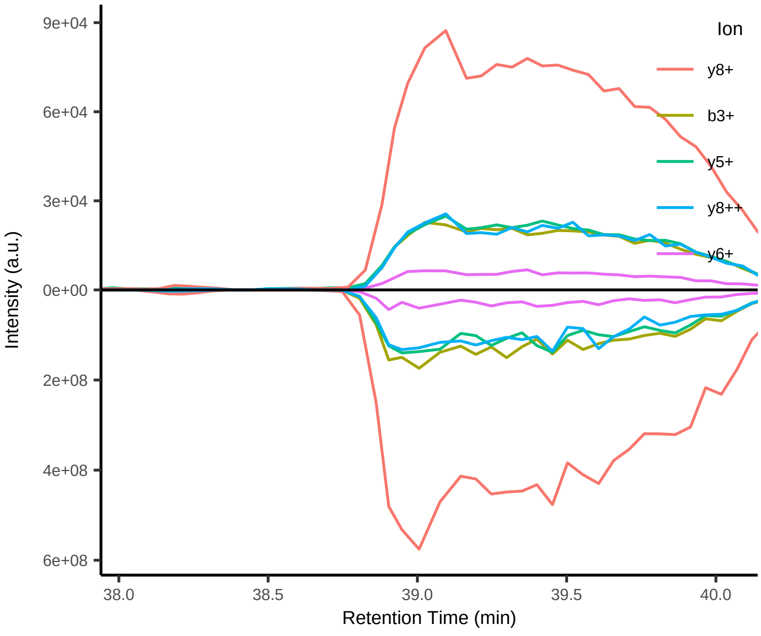

#75 TTV[+6.013809]PFTSW+

Peptide mix
Top 4 transitions: dotp = 0.96; l:h ppm = 161

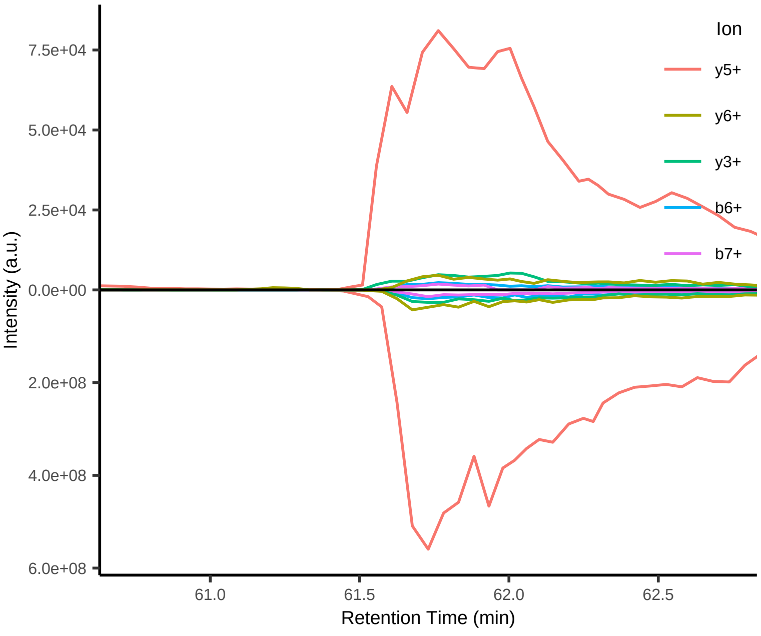

#76 GTTV[+6.013809]PFTSW++

Peptide mix
Top 4 transitions: dotp = 0.93; l:h ppm = 160

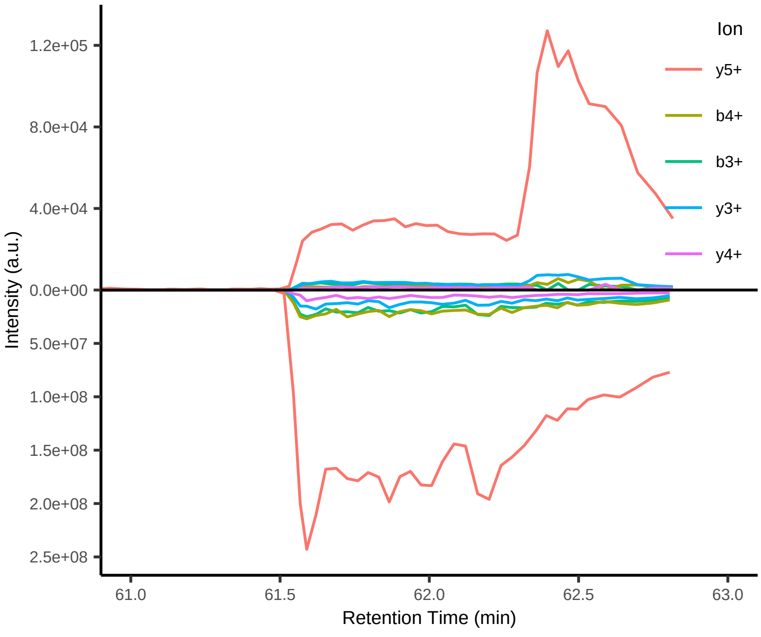

#77 SV[+6.013809]LTAFLM[+15.994915]L++

Peptide mix
Top 4 transitions: dotp = 0.99; l:h ppm = 146

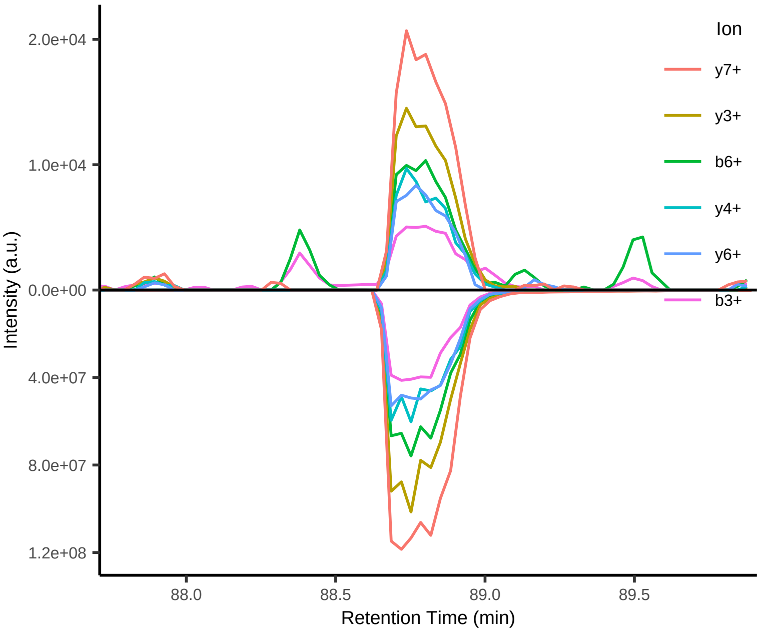

#78 RFGTTV[+6.013809]PFTSW++

Peptide mix
Top 4 transitions: dotp = 0.88; l:h ppm = 144

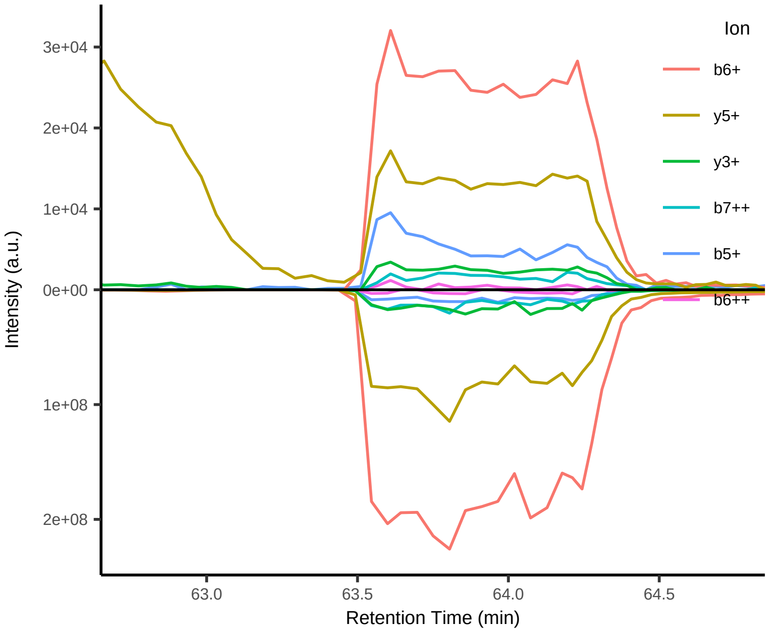

#79 LMLWL[+7.017164]PHW++

Peptide mix
Top 4 transitions: dotp = 0.95; l:h ppm = 140

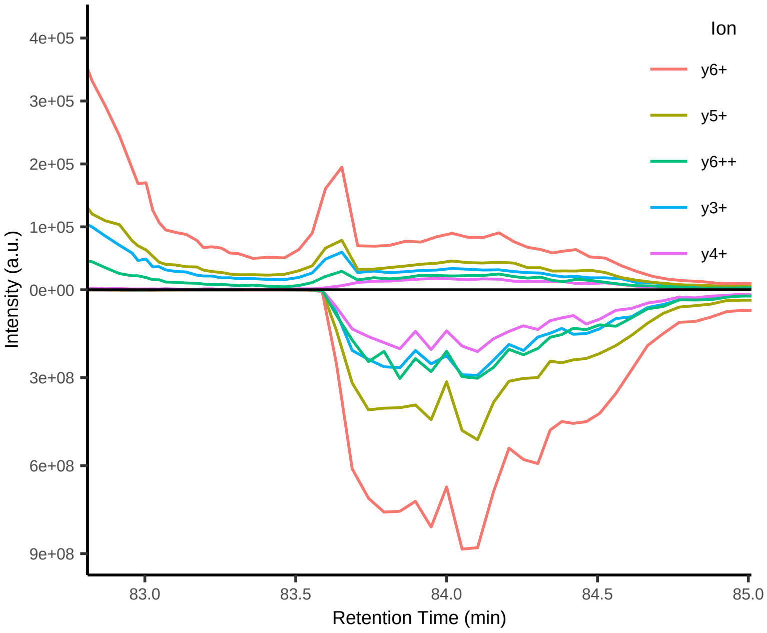

#80 MLWLPHWGL[+7.017164]Y++

Peptide mix
Top 4 transitions: dotp = 0.91; l:h ppm = 139

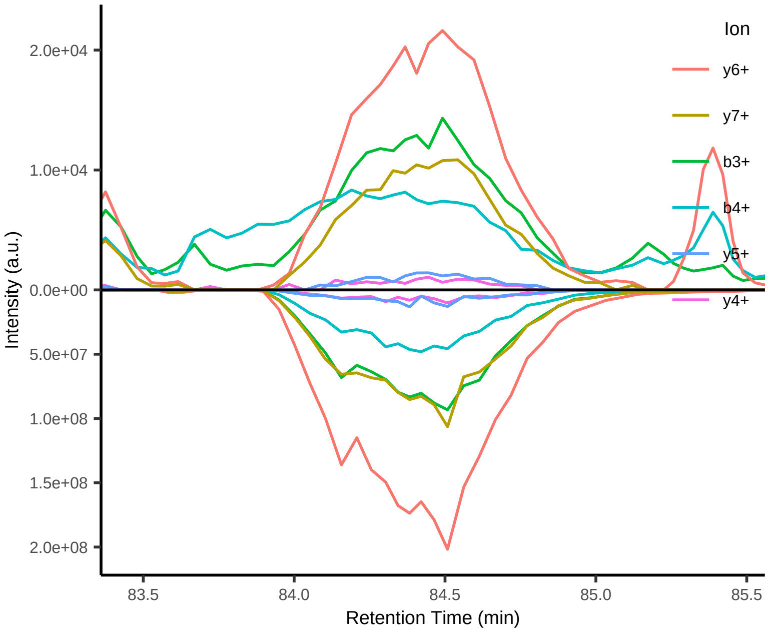

#81 KSV[+6.013809]LTAFLM[+15.994915]L++

Peptide mix
Top 4 transitions: dotp = 0.96; l:h ppm = 130

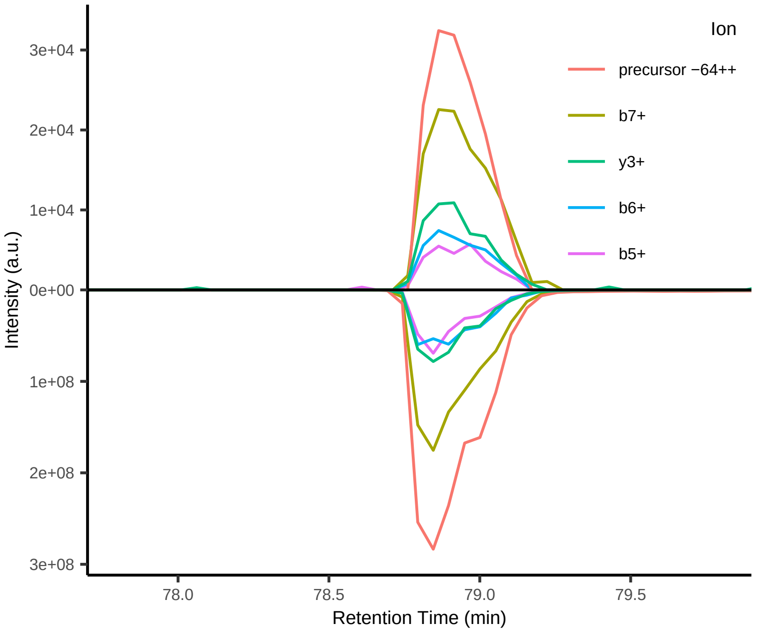

#82 M[+15.994915]LWLPHWGL[+7.017164]Y++

Peptide mix
Top 4 transitions: dotp = 0.94; l:h ppm = 127

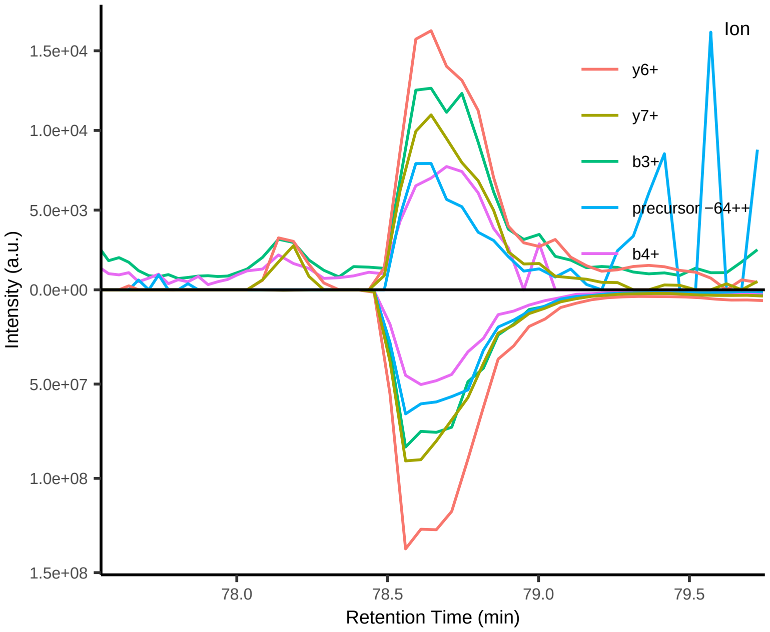

#83 GKSTL[+7.017164]VRLL+++

Peptide mix
Top 4 transitions: dotp = 0.97; l:h ppm = 131

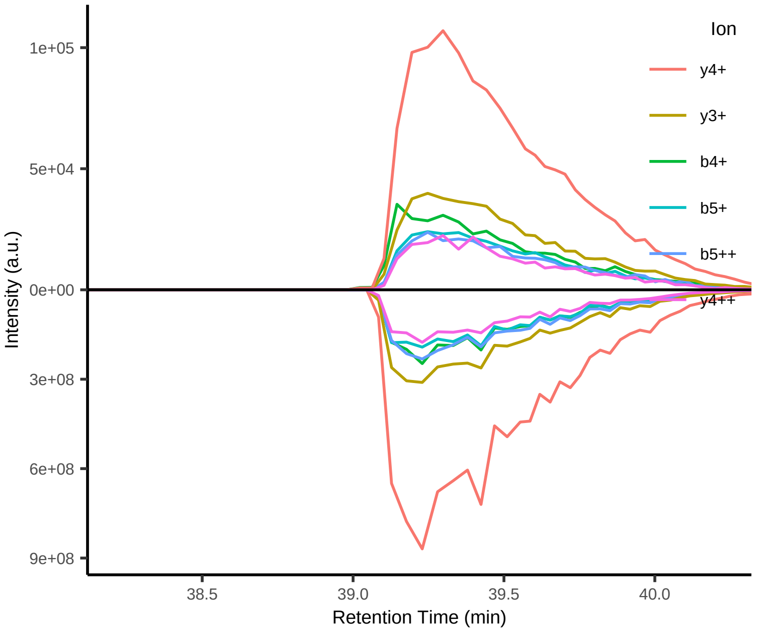

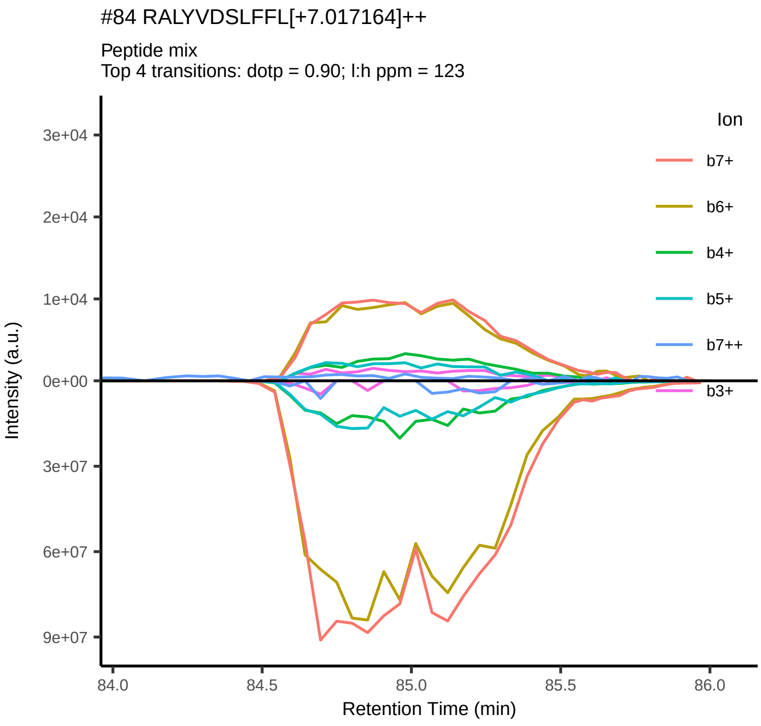
#84 RALYVDSLFFL[+7.017164]++
Peptide mix
Top 4 transitions: dotp = 0.90; l:h ppm = 123
Ion
b7+
b6+
b4+
b5+
b7++
b3+
3e+04
2e+04
1e+04
0e+00
3e+07
6e+07
9e+07
Intensity (a.u.)
84.0
84.5
85.0
85.5
86.0
Retention Time (min)

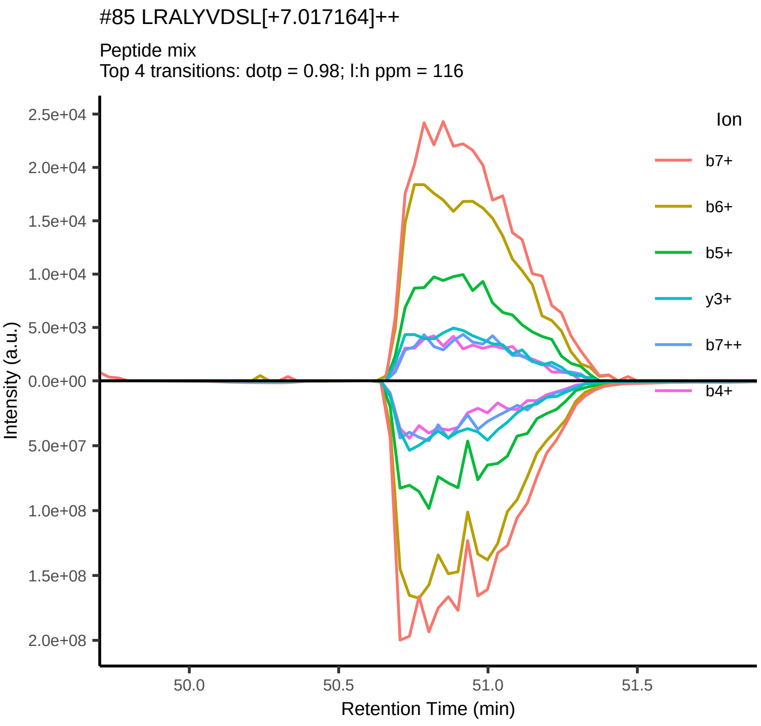
#85 LRALYVDSL[+7.017164]++
Peptide mix
Top 4 transitions: dotp = 0.98; l:h ppm = 116
Ion
b7+
b6+
b5+
y3+
b7++
b4+
2.5e+04
2.0e+04
1.5e+04
1.0e+04
5.0e+03
0.0e+00
5.0e+07
1.0e+08
1.5e+08
2.0e+08
Intensity (a.u.)
50.0
50.5
51.0
51.5
Retention Time (min)

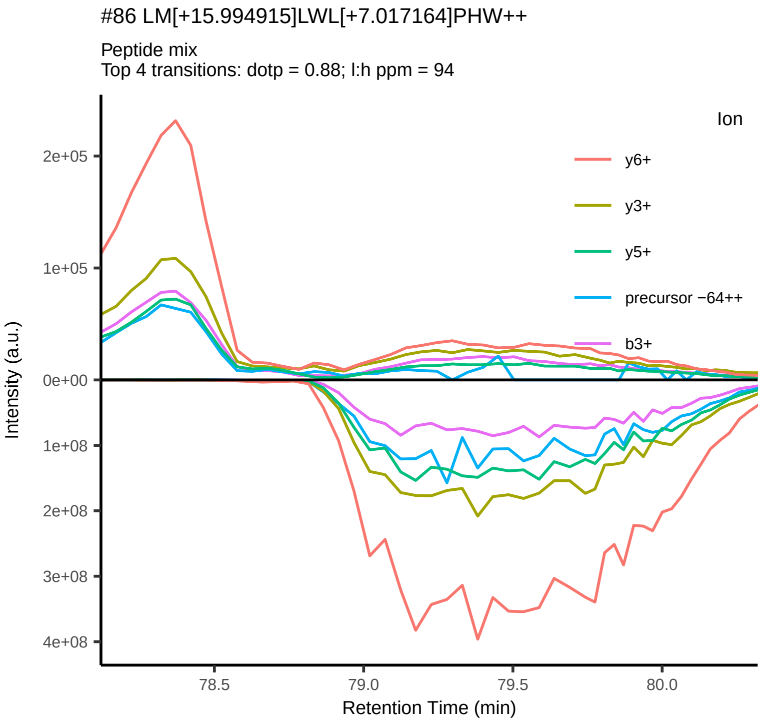
#86 LM[+15.994915]LWL[+7.017164]PHW++
Peptide mix
Top 4 transitions: dotp = 0.88; l:h ppm = 94
Ion
y6+
y3+
y5+
precursor −64++
b3+
2e+05
1e+05
0e+00
1e+08
2e+08
3e+08
4e+08
Intensity (a.u.)
78.5
79.0
79.5
80.0
Retention Time (min)

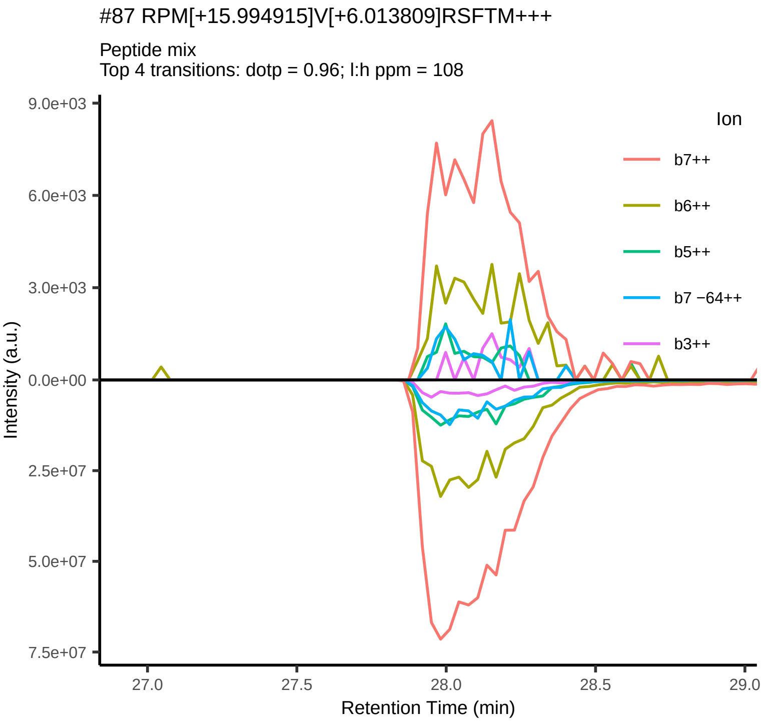
#87 RPM[+15.994915]V[+6.013809]RSFTM+++
Peptide mix
Top 4 transitions: dotp = 0.96; l:h ppm = 108
Ion
b7++
b6++
b5++
b7 −64++
b3++
9.0e+03
6.0e+03
3.0e+03
0.0e+00
2.5e+07
5.0e+07
7.5e+07
Intensity (a.u.)
27.0
27.5
28.0
28.5
29.0
Retention Time (min)

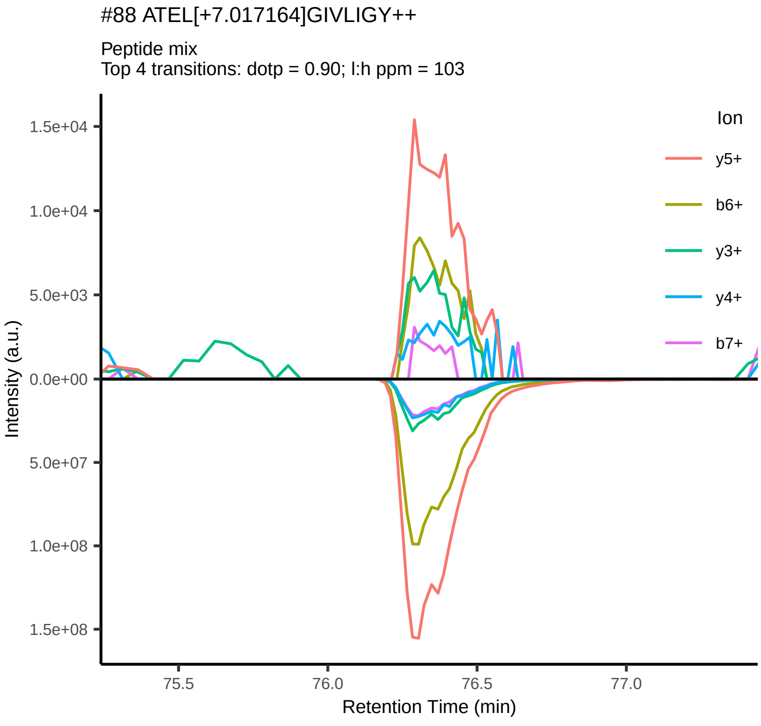
#88 ATEL[+7.017164]GIVLIGY++
Peptide mix
Top 4 transitions: dotp = 0.90; l:h ppm = 103
Ion
y5+
b6+
y3+
y4+
b7+
1.5e+04
1.0e+04
5.0e+03
0.0e+00
5.0e+07
1.0e+08
1.5e+08
Intensity (a.u.)
75.5
76.0
76.5
77.0
Retention Time (min)

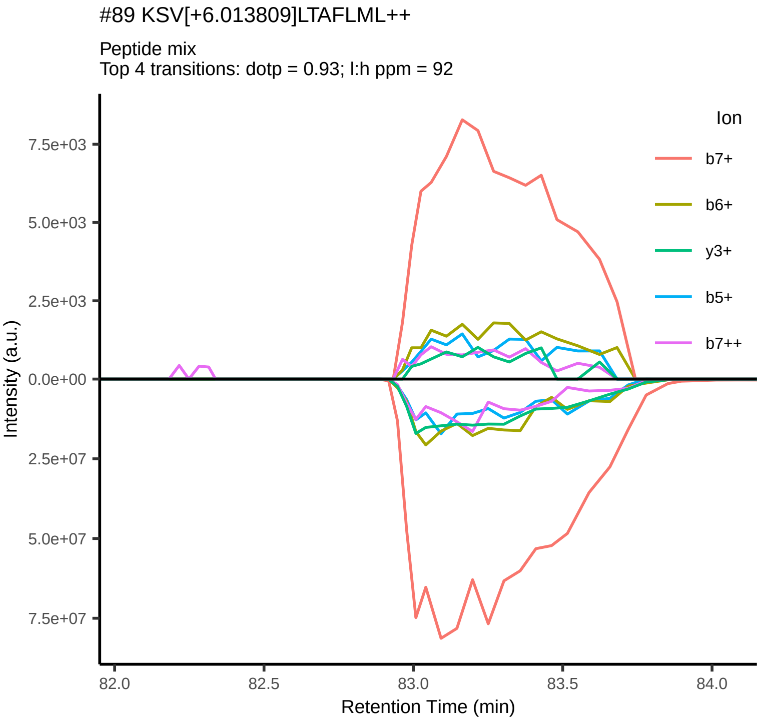
#89 KSV[+6.013809]LTAFLML++
Peptide mix
Top 4 transitions: dotp = 0.93; l:h ppm = 92
Ion
b7+
b6+
y3+
b5+
b7++
7.5e+03
5.0e+03
2.5e+03
0.0e+00
2.5e+07
5.0e+07
7.5e+07
Intensity (a.u.)
82.0
82.5
83.0
83.5
84.0
Retention Time (min)

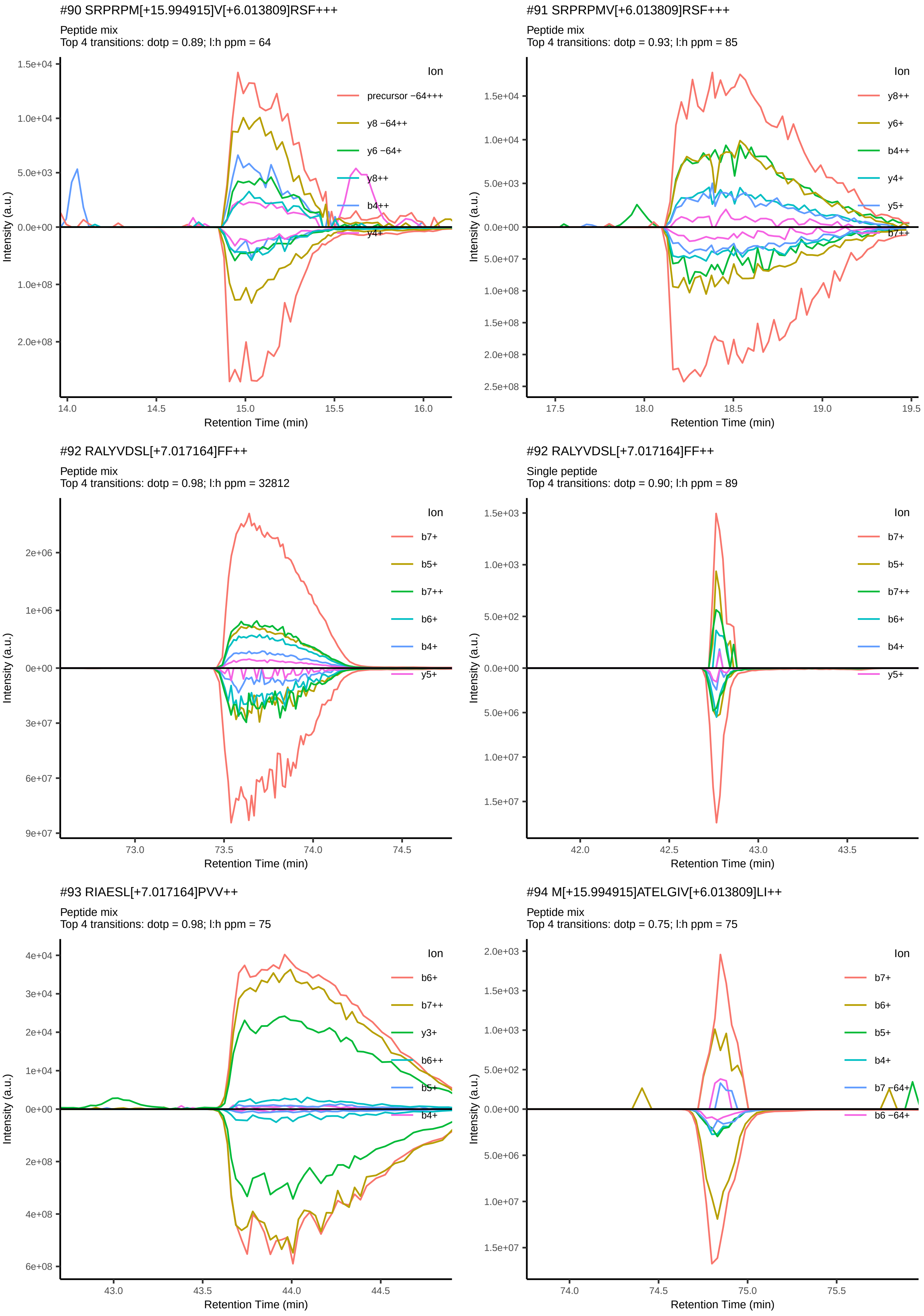
#90 SRPRPM[+15.994915]V[+6.013809]RSF+++
Peptide mix
Top 4 transitions: dotp = 0.89; l:h ppm = 64
Ion
precursor −64+++
y8 −64++
y6 −64+
y8++
b4++
y4+
Intensity (a.u.)
Retention Time (min)
#91 SRPRPMV[+6.013809]RSF+++
Peptide mix
Top 4 transitions: dotp = 0.93; l:h ppm = 85
Ion
y8++
y6+
b4++
y4+
y5+
b7++
Intensity (a.u.)
Retention Time (min)
#92 RALYVDSL[+7.017164]FF++
Peptide mix
Top 4 transitions: dotp = 0.98; l:h ppm = 32812
Ion
b7+
b5+
b7++
b6+
b4+
y5+
Intensity (a.u.)
Retention Time (min)
#92 RALYVDSL[+7.017164]FF++
Single peptide
Top 4 transitions: dotp = 0.90; l:h ppm = 89
Ion
b7+
b5+
b7++
b6+
b4+
y5+
Intensity (a.u.)
Retention Time (min)
#93 RIAESL[+7.017164]PVV++
Peptide mix
Top 4 transitions: dotp = 0.98; l:h ppm = 75
Ion
b6+
b7++
y3+
b6++
b5+
b4+
Intensity (a.u.)
Retention Time (min)
#94 M[+15.994915]ATELGIV[+6.013809]LI++
Peptide mix
Top 4 transitions: dotp = 0.75; l:h ppm = 75
Ion
b7+
b6+
b5+
b4+
b7 −64+
b6 −64+
Intensity (a.u.)
Retention Time (min)

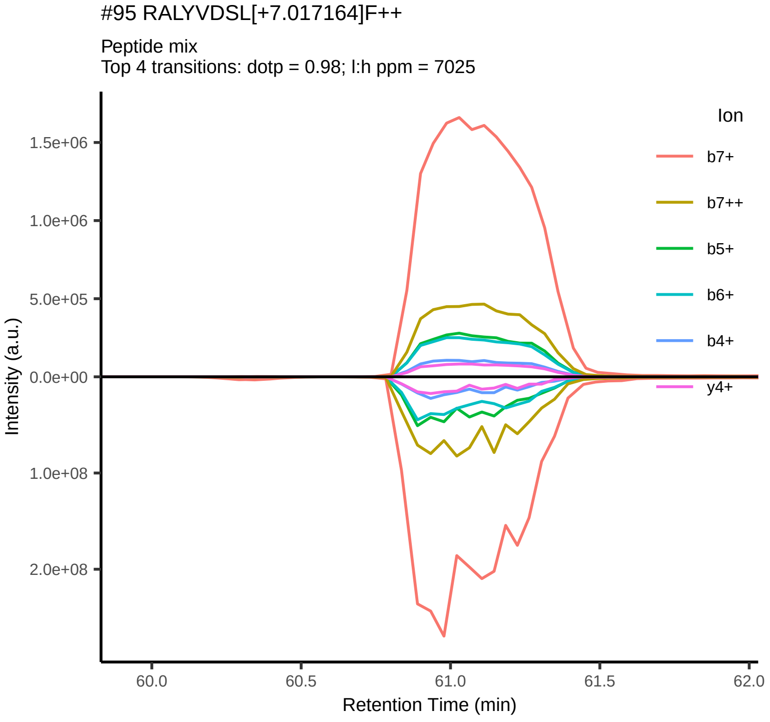
#95 RALYVDSL[+7.017164]F++
Peptide mix
Top 4 transitions: dotp = 0.98; l:h ppm = 7025
Ion
b7+
b7++
b5+
b6+
b4+
y4+
Intensity (a.u.)
Retention Time (min)

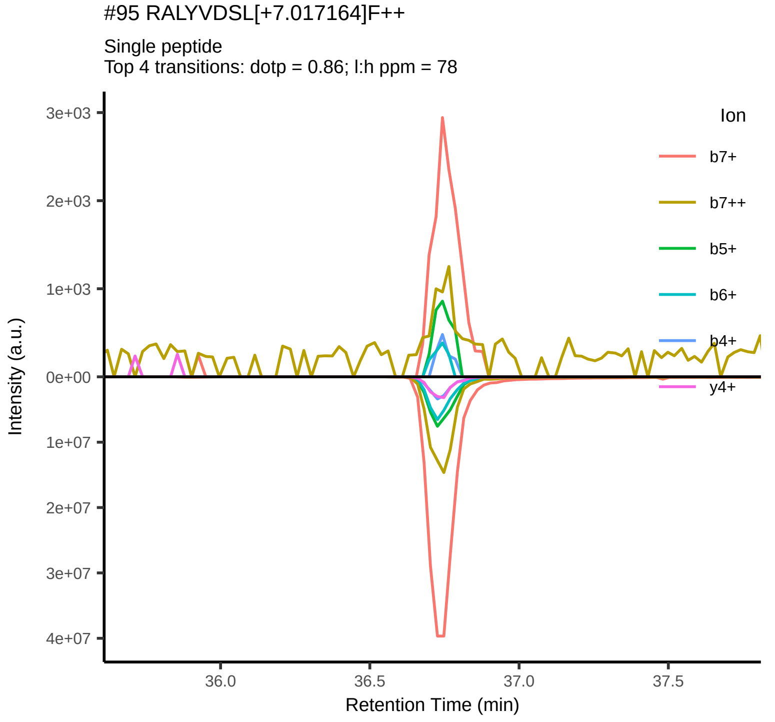
#95 RALYVDSL[+7.017164]F++
Single peptide
Top 4 transitions: dotp = 0.86; l:h ppm = 78
Ion
b7+
b7++
b5+
b6+
b4+
y4+
Intensity (a.u.)
Retention Time (min)

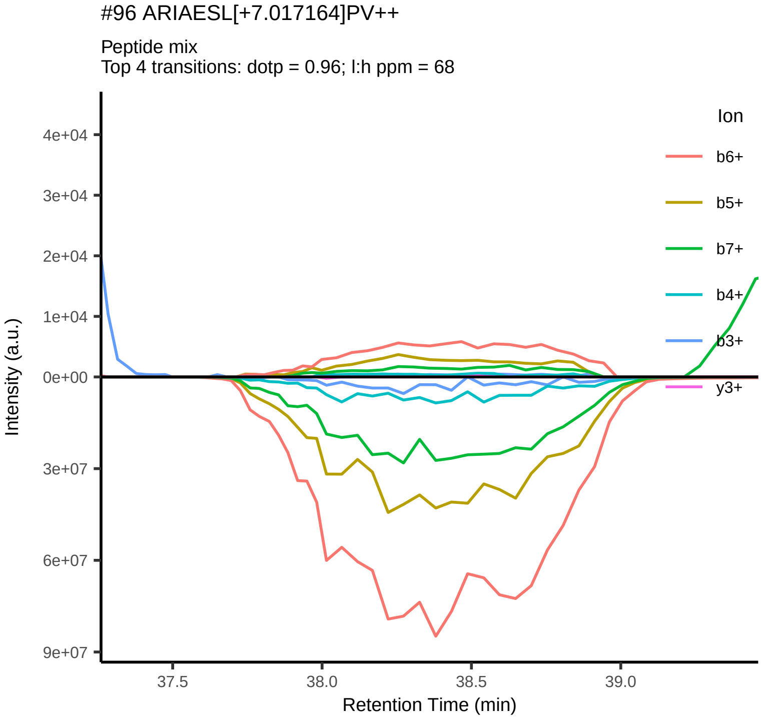
#96 ARIAESL[+7.017164]PV++
Peptide mix
Top 4 transitions: dotp = 0.96; l:h ppm = 68
Ion
b6+
b5+
b7+
b4+
b3+
y3+
Intensity (a.u.)
Retention Time (min)

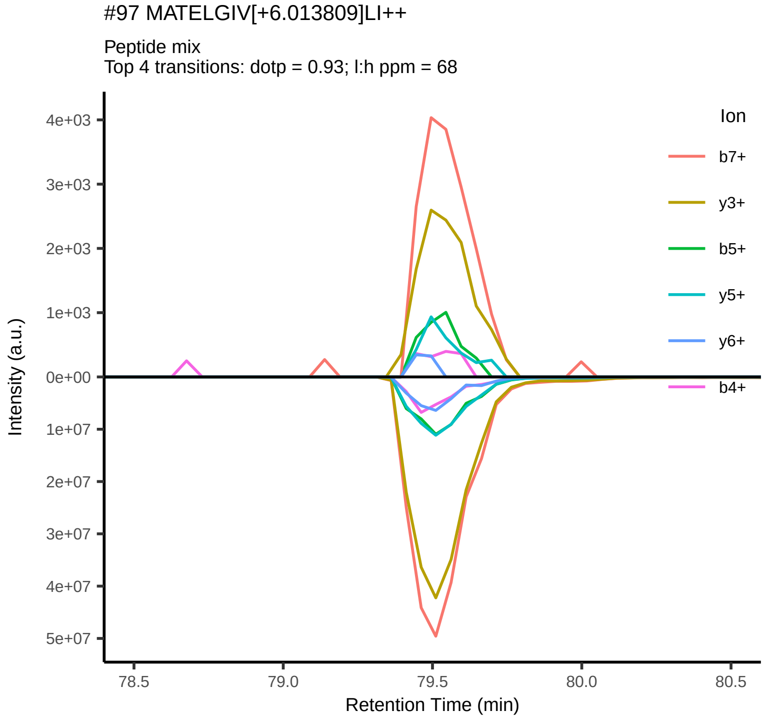
#97 MATELGIV[+6.013809]LI++
Peptide mix
Top 4 transitions: dotp = 0.93; l:h ppm = 68
Ion
b7+
y3+
b5+
y5+
y6+
b4+
Intensity (a.u.)
Retention Time (min)

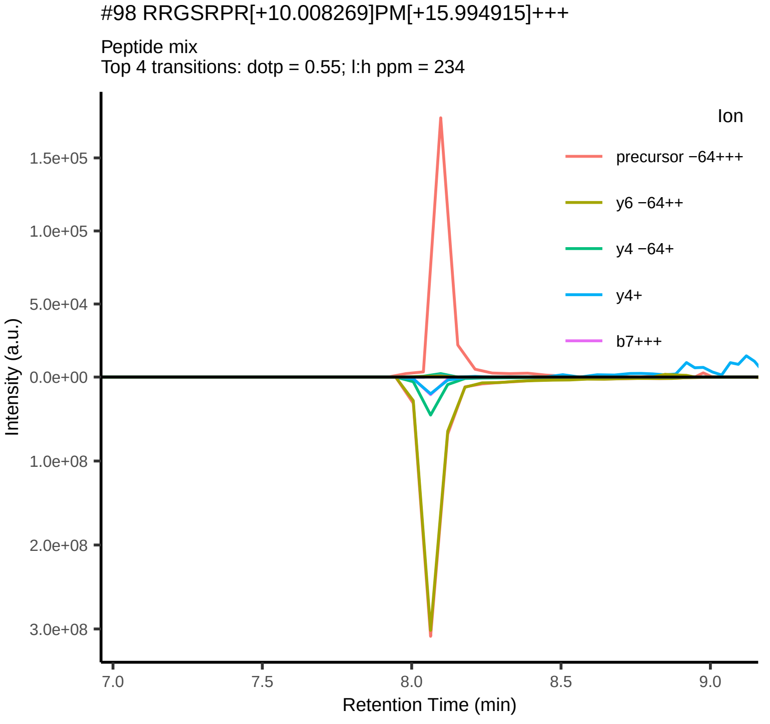
#98 RRGSRPR[+10.008269]PM[+15.994915]+++
Peptide mix
Top 4 transitions: dotp = 0.55; l:h ppm = 234
Ion
precursor −64+++
y6 −64++
y4 −64+
y4+
b7+++
Intensity (a.u.)
Retention Time (min)

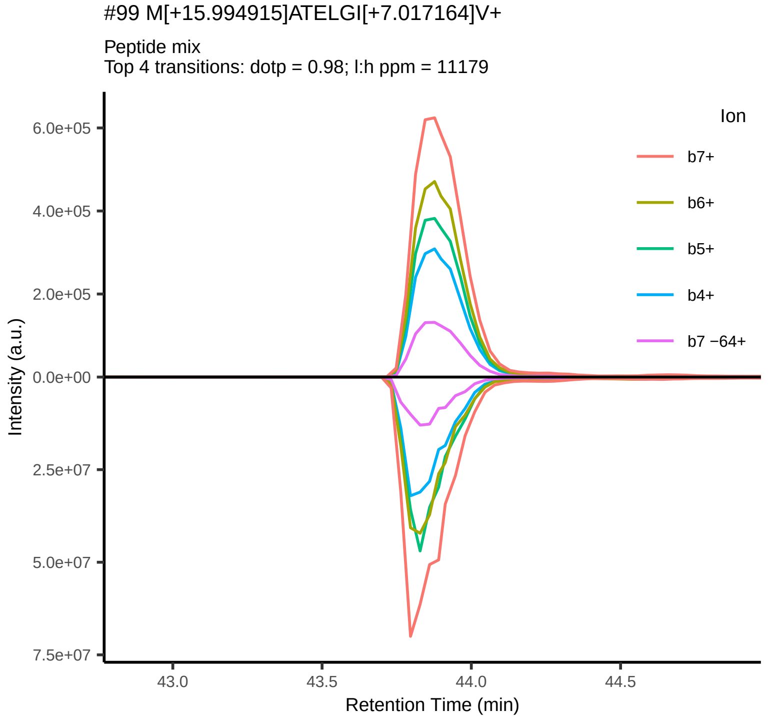
#99 M[+15.994915]ATELGI[+7.017164]V+
Peptide mix
Top 4 transitions: dotp = 0.98; l:h ppm = 11179
Ion
b7+
b6+
b5+
b4+
b7 −64+
Intensity (a.u.)
Retention Time (min)

#99 M[+15.994915]ATELGI[+7.017164]V+

Single peptide
Top 4 transitions: dotp = 0.78; l:h ppm = 8

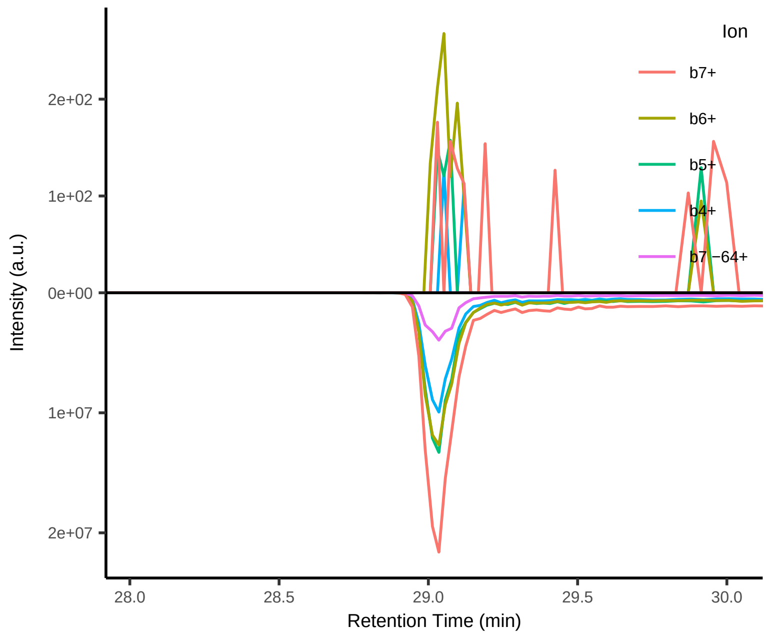

#100 MATELGI[+7.017164]V+

Peptide mix
Top 4 transitions: dotp = 0.97; l:h ppm = 7988

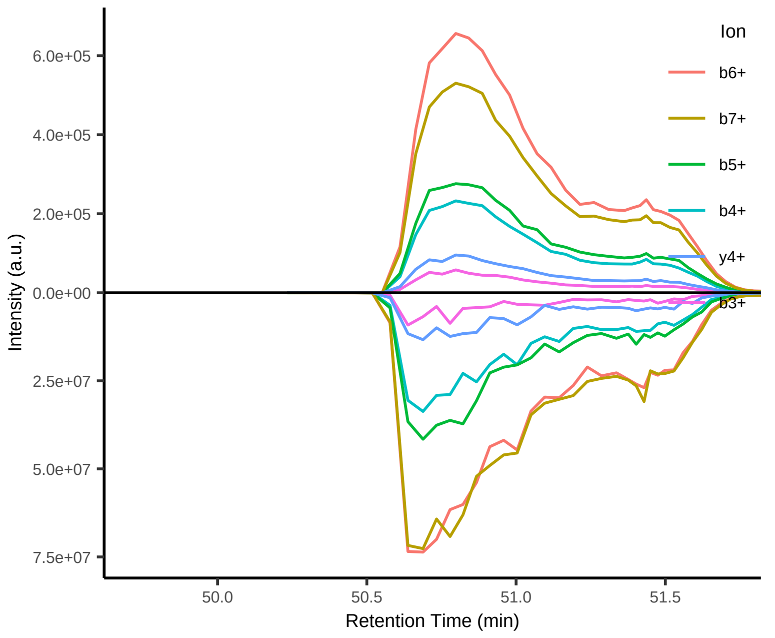

#100 MATELGI[+7.017164]V+

Single peptide
Top 4 transitions: dotp = 0.80; l:h ppm = 7

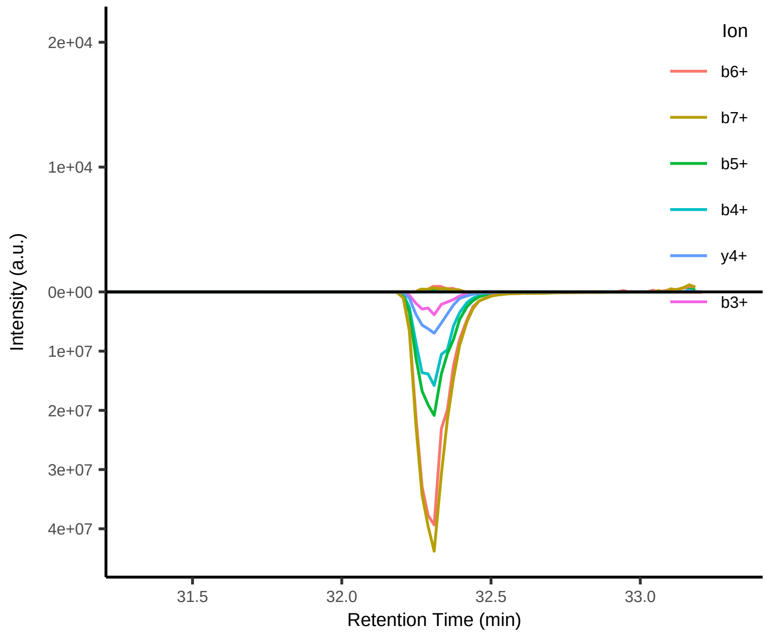

#101 GTTV[+6.013809]PFTSWK[+8.014199]++

Peptide mix
Top 4 transitions: dotp = NA; l:h ppm = NA

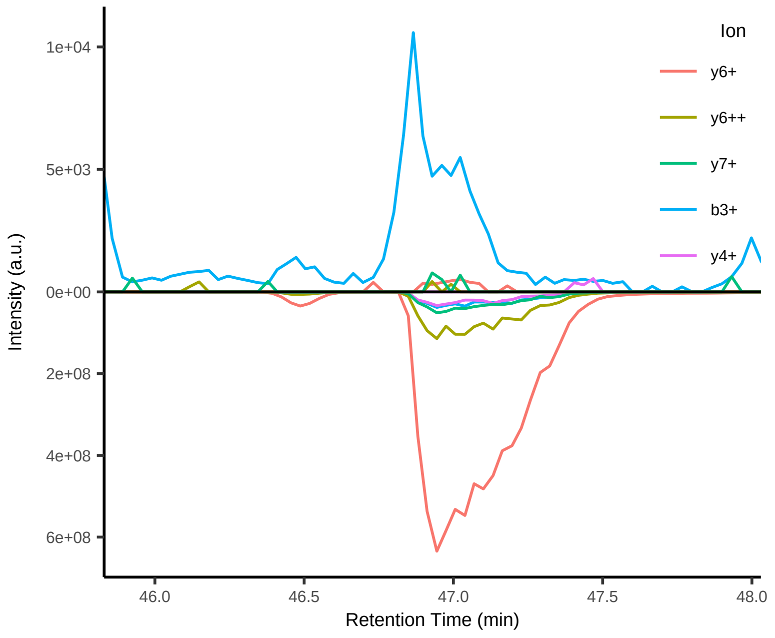

#102 FLM[+15.994915]LWL[+7.017164]PHWGL[+7.017164]++

Peptide mix
Top 4 transitions: dotp = NA; l:h ppm = NA

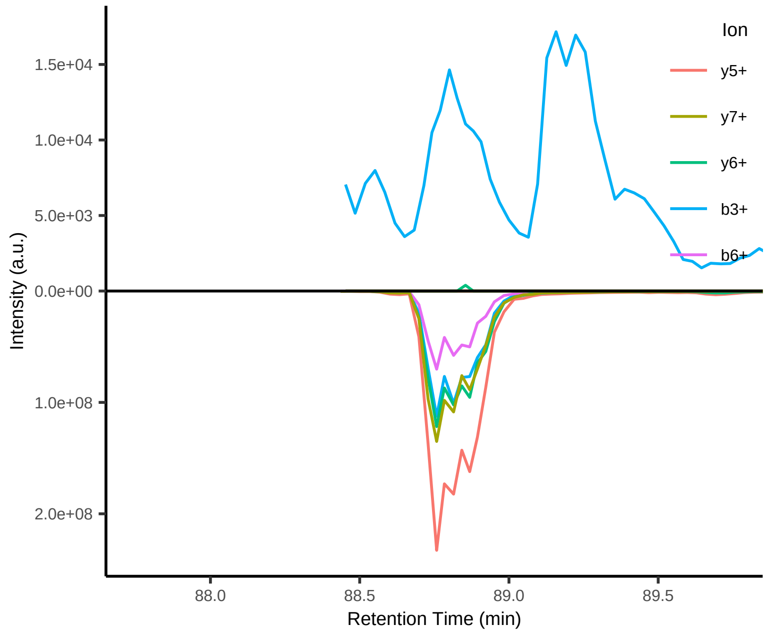

#103 KSV[+6.013809]LTAFL[+7.017164]M[+15.994915]++

Peptide mix
Top 4 transitions: dotp = 0.99; l:h ppm = 453

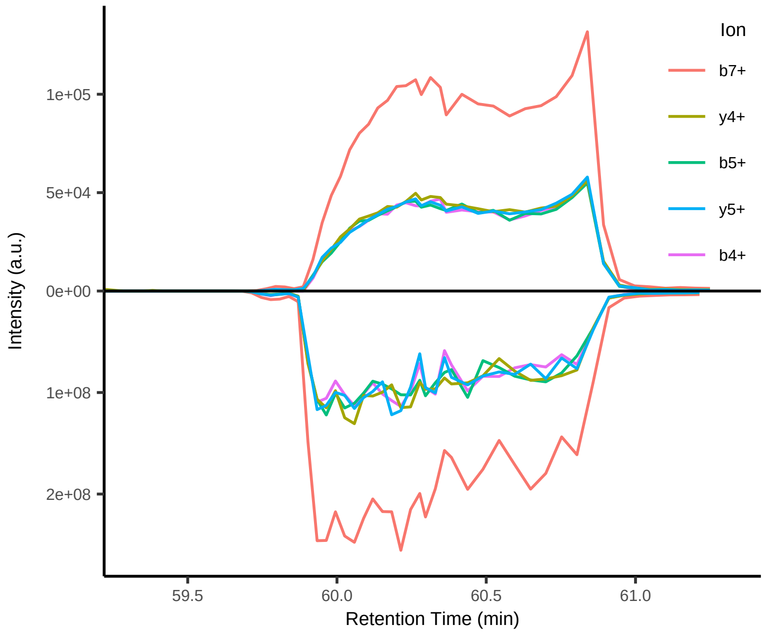

#103 KSV[+6.013809]LTAFL[+7.017164]M[+15.994915]++

Single peptide
Top 4 transitions: dotp = NA; l:h ppm = NA

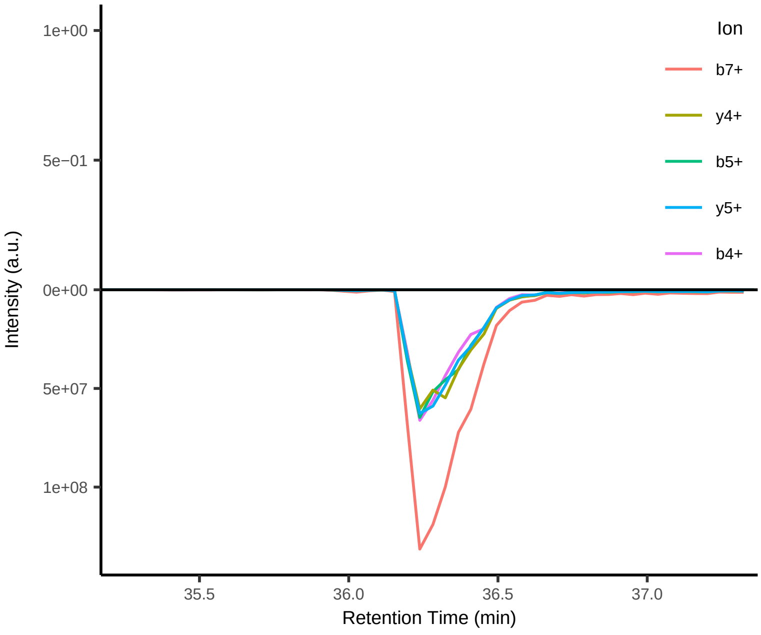

#104 STLVRLLFR[+10.008269]FY+++

Peptide mix
Top 4 transitions: dotp = NA; l:h ppm = NA

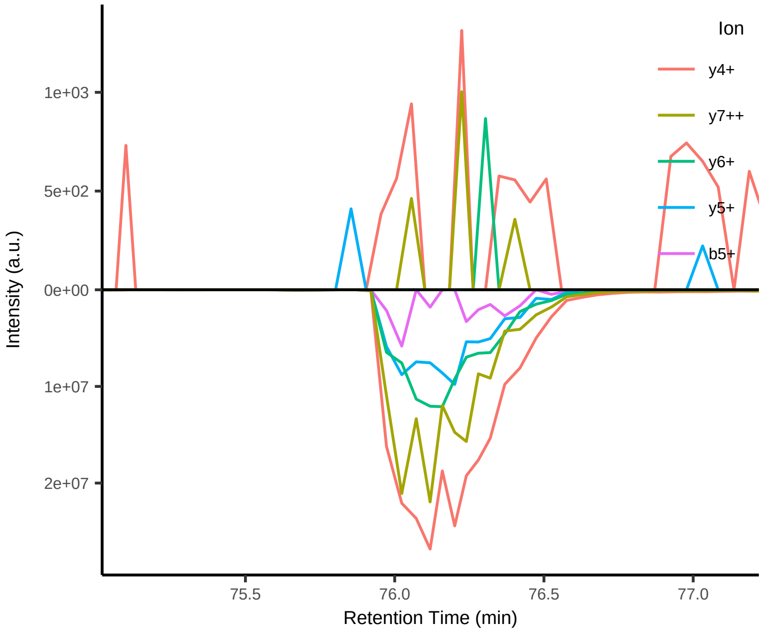

#105 STLV[+6.013809]RLLFRF+++

Peptide mix
Top 4 transitions: dotp = NA; l:h ppm = NA

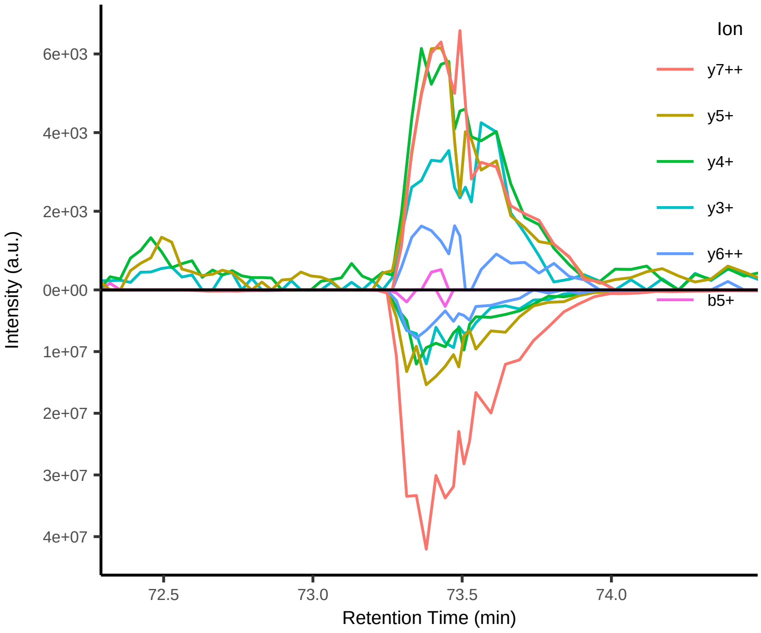

#106 RRGSRPR[+10.008269]PM+++

Peptide mix
Top 4 transitions: dotp = NA; l:h ppm = NA

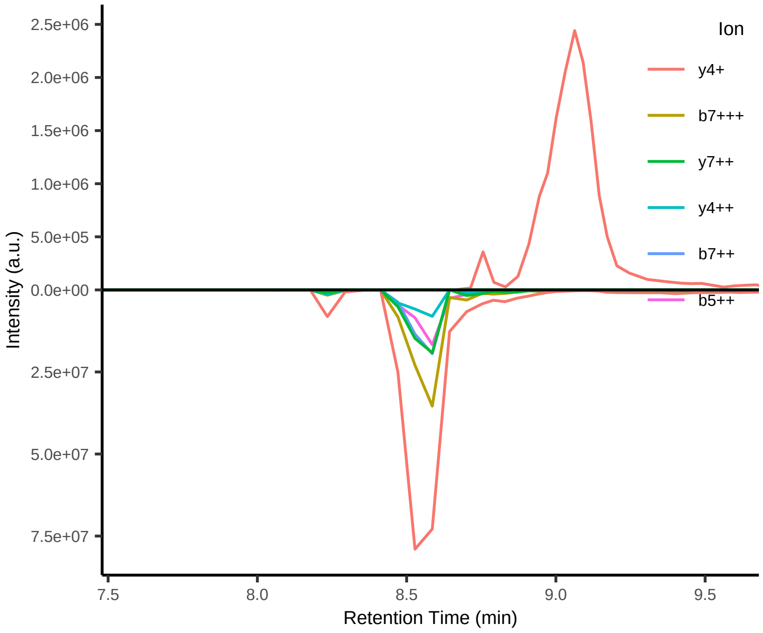

#107 VLIGYFTLV[+6.013809]++

Peptide mix
Top 4 transitions: dotp = NA; l:h ppm = NA

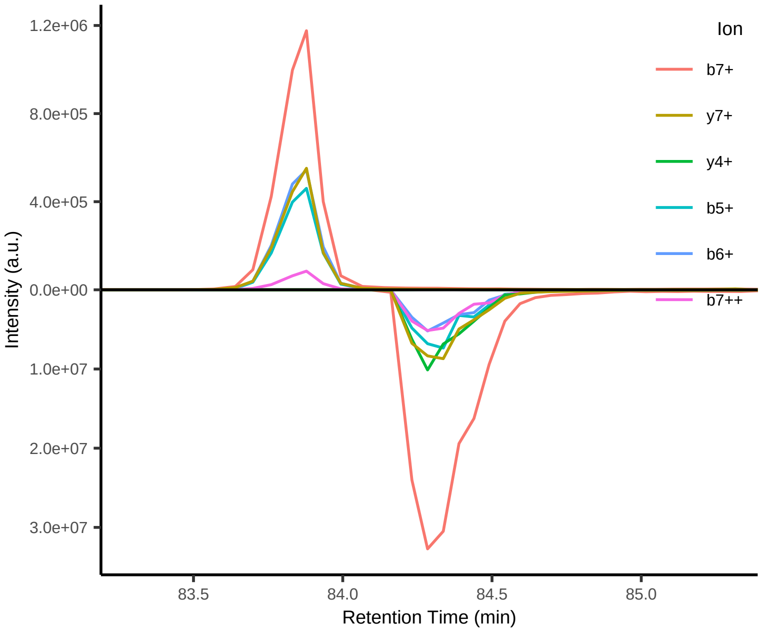

#108 M[+15.994915]ATELGIVL[+7.017164]+

Peptide mix
Top 4 transitions: dotp = NA; l:h ppm = NA

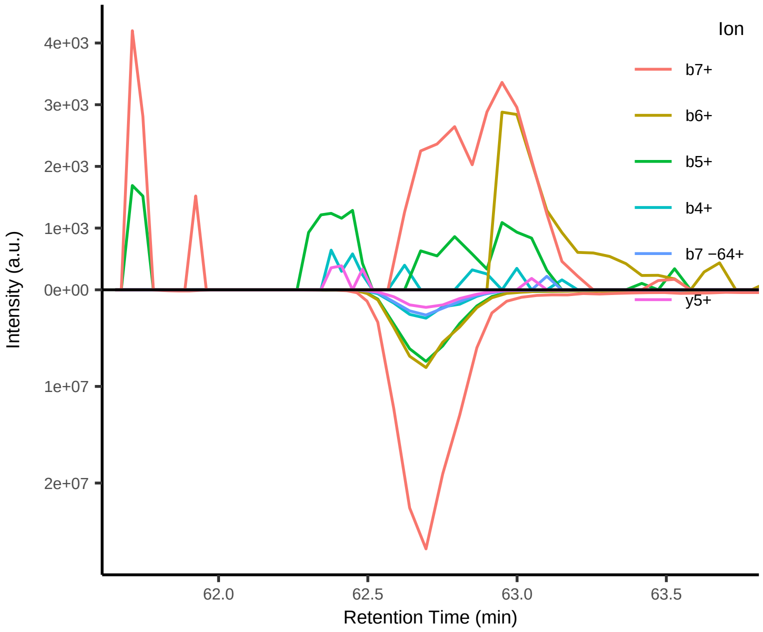

#109 ARIAESL[+7.017164]PVV++

Peptide mix
Top 4 transitions: dotp = NA; l:h ppm = NA

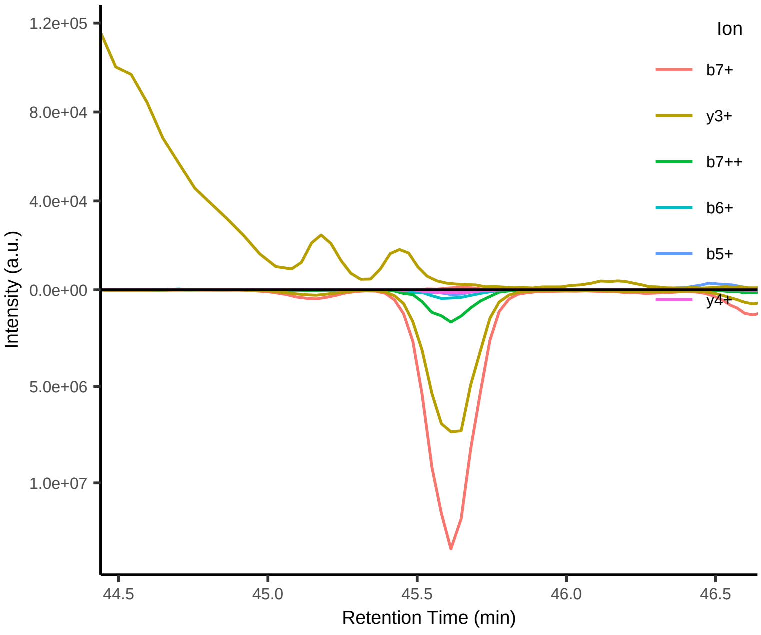

#110 FTGATII[+7.017164]EEY++

Peptide mix
Top 4 transitions: dotp = NA; l:h ppm = NA

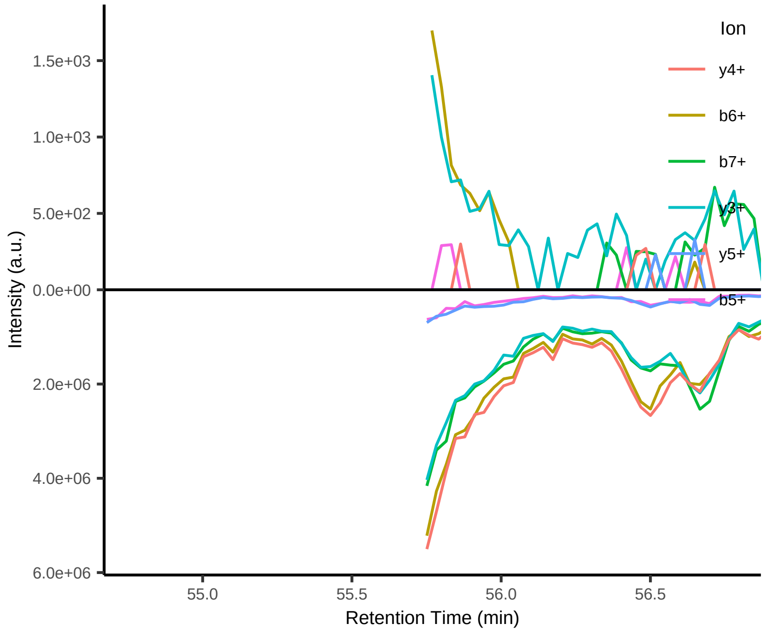

#111 SGSGKSTL[+7.017164]V++

Peptide mix
Top 4 transitions: dotp = NA; l:h ppm = NA

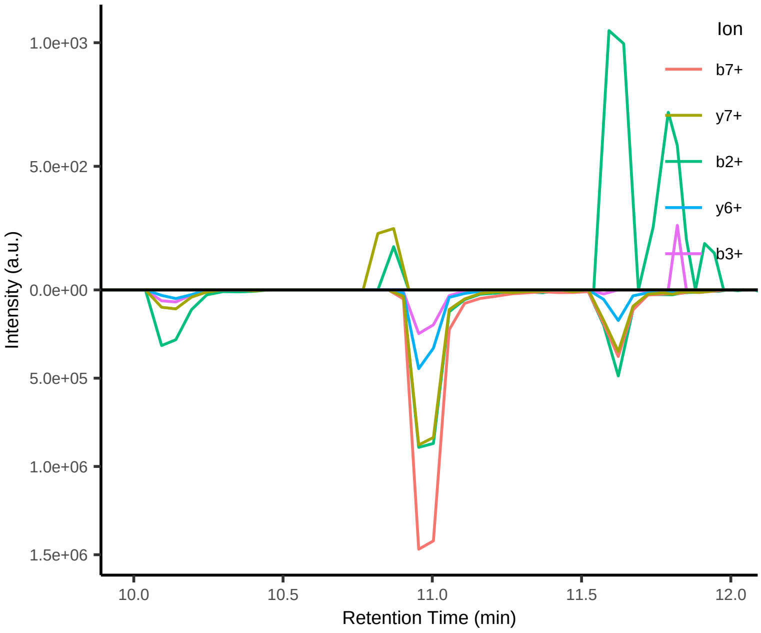

#112 SV[+6.013809]LTAFLML++

Peptide mix
Top 4 transitions: dotp = NA; l:h ppm = NA

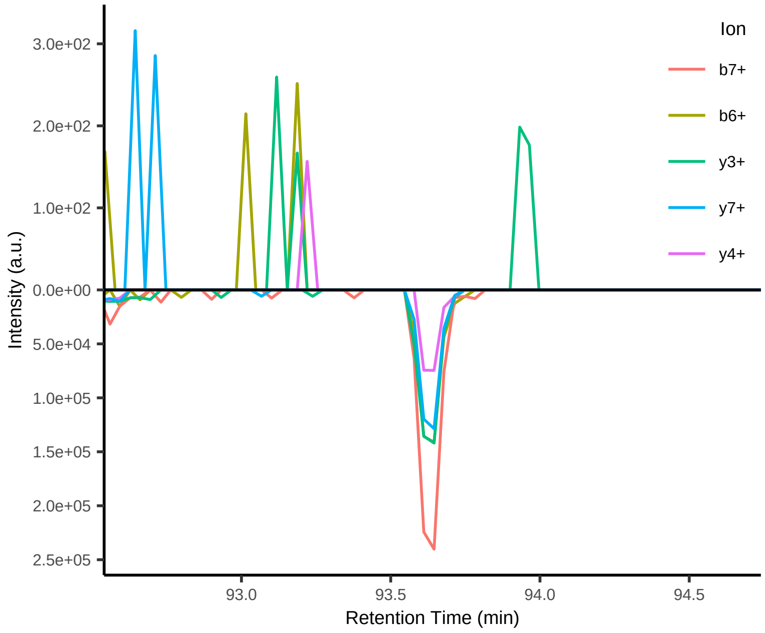

#113 KSV[+6.013809]LTAFL[+7.017164]M++

Peptide mix
Top 4 transitions: dotp = 0.98; l:h ppm = 1350

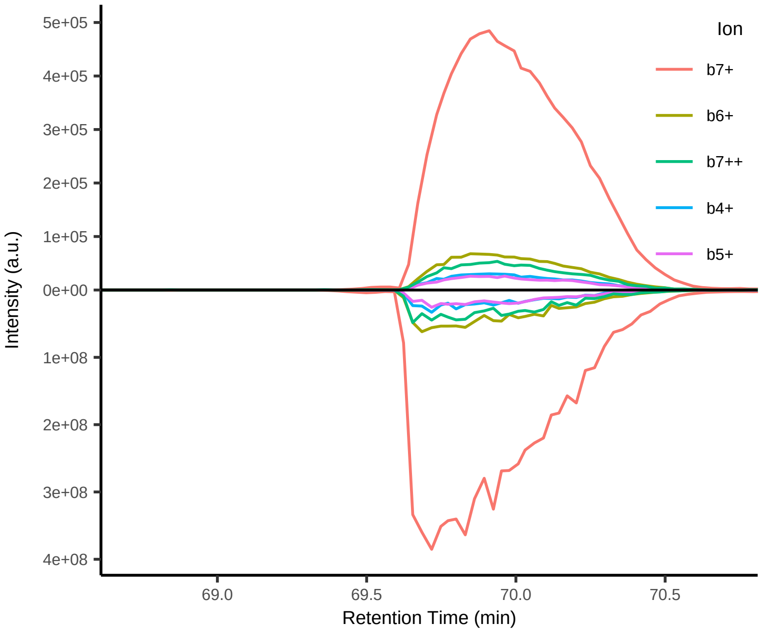

#113 KSV[+6.013809]LTAFL[+7.017164]M++

Single peptide
Top 4 transitions: dotp = NA; l:h ppm = NA

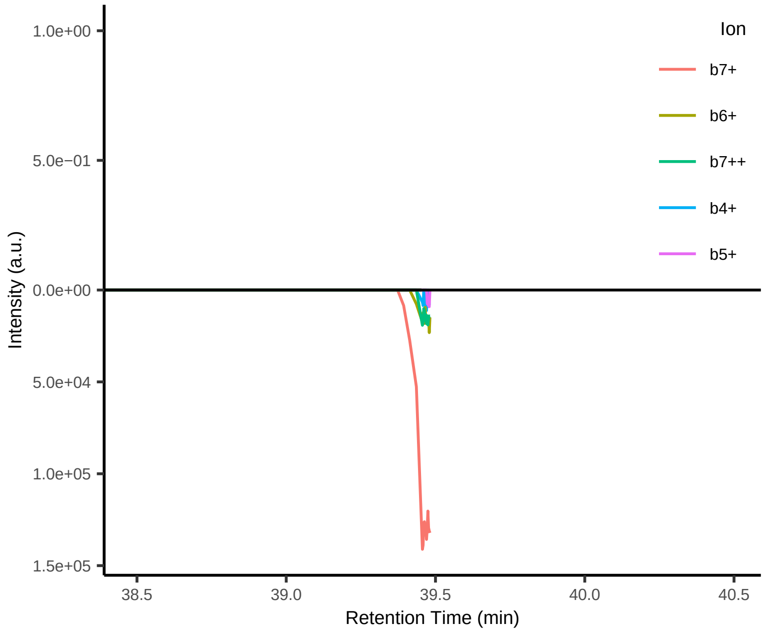

Supplement: Supplementary file 2 — Supplementary file2 (PDF 369 KB) [file 216_2022_3931_MOESM2_ESM.pdf]
